# Accessibility to medical institutions in Aichi Prefecture: A geographic information systems-based analysis

**DOI:** 10.1371/journal.pone.0356748

**Published:** 2026-08-26

**Authors:** Seiichiro Ito, Tomofumi Yamazaki, Kana Sugishita, Koichi Kageyama, Mugita Sato, Megumi Fujita, Satoshi Nakao, Nanaka Ichihara, Yuka Nokura, Moe Yamashita, Reia Hattori, Hirofumi Tamaki, Yoko Ino, Kazuhiro Iguchi, Mitsuhiro Nakamura

**Affiliations:** 1 Laboratory of Drug Informatics, Gifu Pharmaceutical University, Gifu, Japan; 2 Department of Pharmacy, Gifu Prefectural Tajimi Hospital, Tajimi, Gifu, Japan; 3 Department of Pharmacy, Kyushu University Hospital, Fukuoka, Japan; 4 Laboratory of Community Pharmacy, Gifu Pharmaceutical University, Gifu, Japan; 5 Laboratory of Pharmaceutical Health Care and Promotion, Gifu Pharmaceutical University, Gifu, Japan; The Chinese University of Hong Kong, HONG KONG

## Abstract

We investigated the geographic accessibility of medical institutions in Aichi Prefecture, Japan, using Geographic Information Systems (GIS). Three facility types—pharmacies, hospitals, and clinics—were analyzed across 11 secondary healthcare regions. Accessibility was evaluated based on spatial proximity by calculating population and elderly population coverage rates, defined as the proportion of residents located within the vicinity of medical institutions. Catchment areas were defined by travel mode: 400-m buffers represented a 5-min walk, and 2-km buffers approximated a 5-min drive. We tested how results changed when buffer distance varied from 300 m to 3 km. To assess proximity to transit stops and stations, 400-m buffers were generated around bus stops and train stations. Population data at the small-area level (chō-chō-aza units) from the 2020 Census of Japan were overlaid with buffer zones, and areal weighting was applied to estimate populations within buffers. Accessibility indicators were compared across regions and age groups. In the Nagoya–Owari Central region, nearly all residents were covered within 2 km of facilities (pharmacies 100%, hospitals 97%, clinics 100%), whereas the Higashimikawa Northern region had the lowest coverage (pharmacies 59%, hospitals 36%, clinics 72%). Elderly population coverage was lower than total population coverage in rural areas. In urban regions, coverage rates within 400 m of bus stops and train stations reached 96%, whereas in rural regions, they declined to below 50%. Overall, urban areas benefit from concentrated healthcare resources and extensive transit networks, whereas rural and mountainous regions remain underserved. Private-vehicle use may expand potential geographic reach for some residents; however, this does not demonstrate effective access to healthcare, particularly among older adults with mobility limitations. High proximity-based coverage does not imply timely or adequate service utilization. Geographic and demographic perspectives must be integrated into healthcare policy to support community care and sustainable resource allocation under population aging.

## Introduction

Accessibility to medical institutions represents a vital factor for local residents to maintain daily health, enable the early detection of diseases, and receive timely treatment. In recent years, populations have increasingly concentrated in urban areas, whereas rural regions have experienced rapid population aging [1]. As a result, medical resources have increasingly concentrated in urban areas, creating visible disparities in the availability and variety of medical services across regions, particularly in rural areas. This has emerged as a global issue [2].

In Japan, the ongoing demographic shift toward an aging population has generated a growing demand for healthcare services. This issue is especially acute in rural regions, where population decline and the uneven distribution of healthcare resources continue to progress [3]. The capability of residents to appropriately access necessary medical services has become a pressing concern. In countries such as Canada and Australia, where population aging is advancing similarly to Japan and where rural and remote areas face persistent challenges in healthcare access, Krasniuk et al. [4] and Asante et al. [5] conducted interview-based studies with older adults living in rural regions and highlighted the need to improve access to care services. These regional disparities in healthcare accessibility have emerged as a significant challenge in the formulation and implementation of effective health policies.

Several previous studies have examined accessibility to healthcare services. For example, Luo and Wang [6] assessed healthcare accessibility using the Two-Step Floating Catchment Area (2SFCA) method while incorporating the availability of physicians. Additionally, Da Silva et al. [7] applied the 2SFCA method to investigate gaps in access to emergency care in Brazil. In Japan, Kajimoto et al. [8] evaluated population coverage rates of pharmacies by secondary healthcare region and examined pharmacy functions in Wakayama Prefecture. Moreover, Balsa-Barreiro et al. [9] proposed strategic approaches to hospital location planning to better respond to population aging. Additional methodologies used to assess healthcare access include average travel time, distance to the nearest medical institution, and buffer analysis. Among these approaches, spatial proximity-based indicators that quantitatively measure the reachability of medical institutions from places of residence provide useful insights into the actual accessibility experienced by residents.

**Fig 1:**
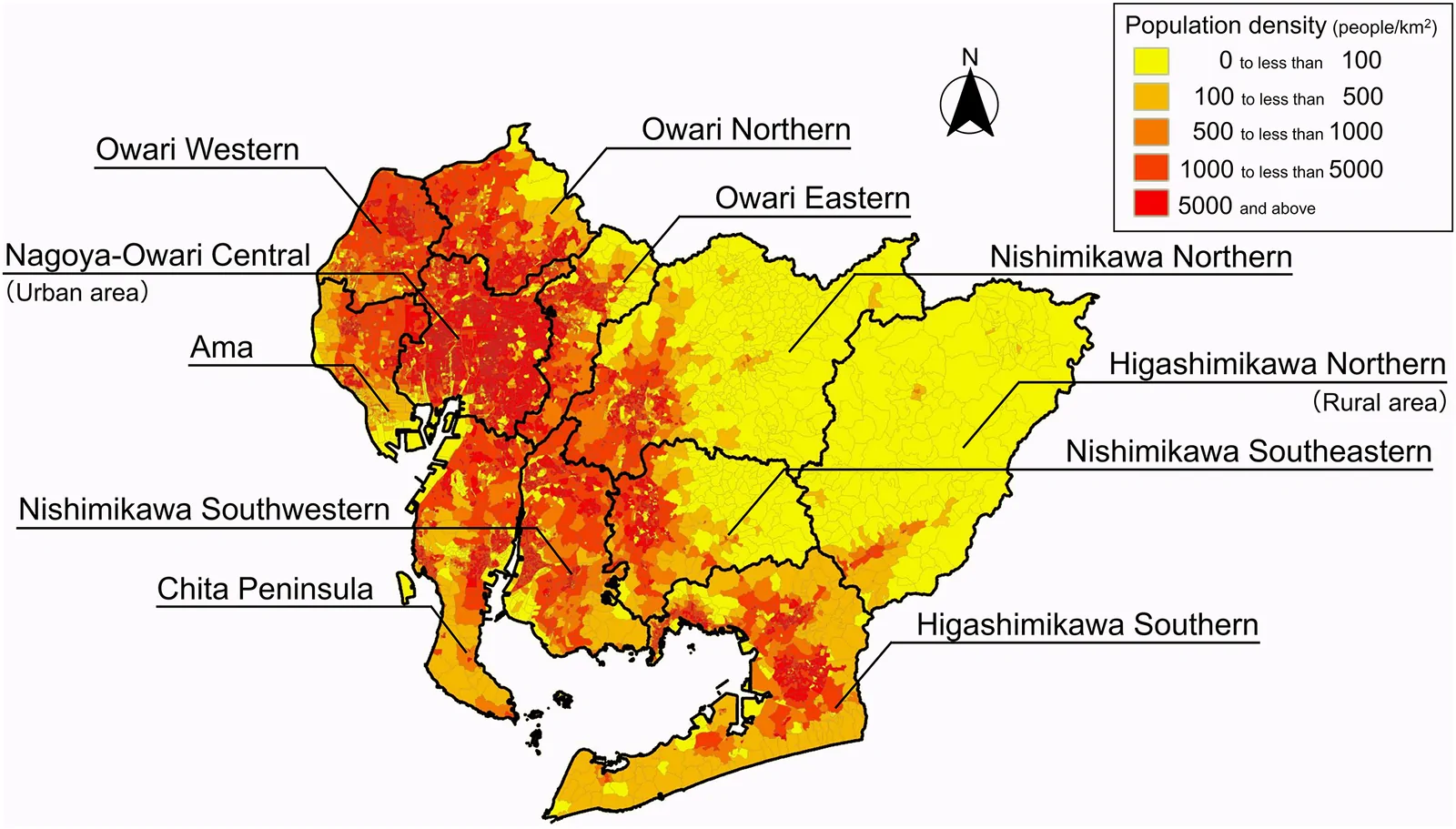
Distribution of population density in Aichi Prefecture. Created in ArcGIS Pro using publicly available data from e-Stat and the National Land Numerical Information Download Site. The source datasets are licensed under CC BY 4.0. The authors processed and visualized the data to produce this figure.

To the best of our knowledge, no previous study has comprehensively assessed accessibility to pharmacies, hospitals, and clinics within a region under both walking- and car-based assumptions. Moreover, there is a lack of prior research using elderly population coverage rates as an accessibility indicator. The aim of this study was to elucidate regional disparities in accessibility to medical institutions in Aichi Prefecture, Japan, a region characterized by both densely populated urban areas and sparsely populated mountainous areas. Aichi Prefecture includes Nagoya City—the third most populous city in Japan outside of Tokyo’s special wards—representing a highly urbanized setting [10]. In contrast, the northeastern part of the prefecture consists of mountainous terrain with low population density (Fig 1). These contrasting geographic and demographic characteristics indicate the potential for substantial differences in healthcare accessibility between urban and rural areas. By focusing on a single prefecture that incorporates both settings, this study provides valuable insights into how geographic diversity within a region affects equity in healthcare access. The findings are expected to support more effective and regionally tailored healthcare policy planning, particularly in the context of population aging and healthcare resource allocation.

Aichi Prefecture covers an area of 5,170 km^2^ and had a population of 7,541,123 in 2020. Its population density was 1,458.6 persons per km^2^, approximately 4.3 times higher than the national average of 338.4 persons per km^2^ [10]. Furthermore, as of March 31, 2020, Aichi Prefecture had the highest number of privately owned automobiles in Japan [11], creating an environment in which access to medical institutions by both walking and private car can be realistically assumed. Considering the geographic and transportation conditions, this study used Geographic Information Systems (GIS) to quantitatively evaluate the spatial distribution of healthcare resources and accessibility to medical institutions across different regions. We formulated and tested the following hypotheses regarding healthcare accessibility. First, population coverage rates of medical institutions are positively correlated with population density. Second, population coverage rates of medical institutions are negatively correlated with the proportion of older adults in the population. Third, the spatial distribution of healthcare resources is associated with regional characteristics. The analysis also examined relationships between accessibility and regional characteristics, such as population density and the aging rate. In particular, buffer analyses were conducted based on assumed catchment areas for walking and driving, combined with small-area population data from the national census. Through this approach, the aim of this study was to visualize the relationship between the distribution of healthcare resources and regional characteristics, providing insights to inform local healthcare policy.

## Materials and methods

### Study region and selected healthcare facilities

To clarify regional disparities in accessibility to medical institutions, this study divided Aichi Prefecture into 11 secondary healthcare regions, based on the Aichi Prefecture Regional Health and Medical Care Plan published in March 2024 [12,13]. These regions included: Nagoya–Owari Central, Owari Northern, Owari Western, Owari Eastern, Ama, Chita Peninsula, Nishimikawa Southwestern, Nishimikawa Southeastern, Higashimikawa Southern, Higashimikawa Northern, and Nishimikawa Northern. Each region possesses distinct characteristics regarding population size, geographic features, transportation infrastructure, and the density of medical institutions (Table 1). For example, the Nagoya–Owari Central region is a highly urbanized area with a high concentration of advanced medical institutions, whereas regions such as Higashimikawa Northern and Nishimikawa Northern have relatively few medical institutions, requiring residents to travel long distances to obtain appropriate healthcare services.

**Table 1:** Demographic and geographic attributes by secondary healthcare region in Aichi Prefecture.

Secondary medical area	Population (n)	Area (km^2^)	Population density (people/km^2^)	Elderly Population (%)
Nagoya-Owari Central	2,499,750	368.4	6,785	24.2
Owari Northern	731,714	296.0	2,472	26.0
Owari Western	513,914	193.2	2,660	27.2
Owari Eastern	478,049	230.1	2,077	24.4
Ama	326,898	208.5	1,568	27.5
Chita Peninsula	625,161	392.2	1,594	24.9
Nishimikawa Southwestern	704,834	363.8	1,938	22.0
Nishimikawa Southeastern	427,932	443.9	964	23.1
Higashimikawa Southern	694,662	671.0	1,035	26.5
Higashimikawa Northern	52,207	1,052.4	50	38.5
Nishimikawa Northern	486,002	950.5	511	22.4

In this study, three types of medical institutions were selected as targets: pharmacies, hospitals, and clinics. The location data for pharmacies were derived from address information listed in the “List of Medical Institutions by Code Classification,” published by the Tokai-Hokuriku Regional Bureau of Health and Welfare. These addresses were converted into geographic coordinates (latitude and longitude) using the “CSV Address Matching Service” provided by the Center for Spatial Information Science at the University of Tokyo [14]. All 3,962 pharmacy addresses were successfully geocoded using the CSV Address Matching Service. These addresses were geocoded with varying levels of spatial precision. Among the 3,628 pharmacies located within Aichi Prefecture, 3,250 (approximately 89.7%) were matched to the most detailed geographic units corresponding to city blocks and individual lot numbers. Additionally, 294 addresses were matched to subdivisions roughly equivalent to neighborhood sections (chōme and kōaza), 79 to larger administrative subdivisions similar to wards or local districts (ōaza), 1 to the ward level within designated cities, and 4 to the broader municipality level (city, town, or village). For the entire dataset of 3,962 pharmacies, 3,525 (about 89.0%) were geocoded at the finest level of city block and lot number, 313 at neighborhood subdivision level, 119 at larger district level, 1 at ward level, and 4 at municipality level. The location data for medical institutions, bus stops, and train stations were obtained from the National Land Numerical Information download service of the Ministry of Land, Infrastructure, Transport and Tourism (MLIT) [15]. The medical institution dataset included hospitals, clinics, and dental clinics; for this study, only hospitals and clinics were extracted for analysis. The sources of all data used for analysis and figure creation in this study are summarized in Table 2.

**Table 2:** Data sources used for analysis and figure creation in this study.

Type	Name	Source	Format	License	Creation date	Acquisition date
Administrative boundary data	Healthcare regions	The National Land Numerical Information download service^*^	JGD2011, WGS, Shapefile	CC BY 4.0	2020	10-04-25
	Small-area	e-Stat^†^	JGD2011, WGS Plane Cartesian Coordinate System, Shapefile	CC BY 4.0	2020	10-04-25
Statistical data	Population by age and sex	e-Stat^†^	CSV	CC BY 4.0	2020	07-05-25
Location information	Pharmacies	List of Medical Institutions by Code Classification^‡^	Table	PDL1.0	01-04-25	10-04-25
	Medical institutions	The National Land Numerical Information download service^*^	JGD2011, WGS, Shapefile	CC BY 4.0	2020	18-04-25
	Bus stops	The National Land Numerical Information download service^*^	JGD2011, WGS, Shapefile	CC BY 4.0	2022	18-04-25
	Railways	The National Land Numerical Information download service^*^	JGD2011, WGS, Shapefile	CC BY 4.0	2023	18-04-25

* The Ministry of Land, Infrastructure, Transport and Tourism (MLIT) [15].

† Portal Site of Official Statistics of Japan [16].

‡ The Tokai-Hokuriku Regional Bureau of Health and Welfare [17].

### Accessibility indicators and analytical methods

To evaluate accessibility to medical institutions, this study focused on spatial proximity and used two key indicators: population coverage rate and elderly population coverage rate. The population coverage rate represented the proportion of the total population within a given area that resided in the vicinity of medical institutions, whereas the elderly population coverage rate indicated the same proportion specifically for individuals aged 65 years and older. In this study, “coverage” was used as a proximity-based measure of potential access (i.e., geographic reachability) rather than a direct measure of the ease of obtaining medical services. Because our analysis did not incorporate healthcare supply capacity (e.g., numbers of physicians, consultation volume, or service availability), high coverage rates (even approaching 100%) do not necessarily imply timely access or adequate service utilization; for example, patients may still experience long waiting times in areas with limited provider capacity despite geographic proximity.

Catchment areas around target facilities were defined based on two travel modes: walking and driving. Accordingly, buffer zones with radii of 400 m and 2 km were generated around each facility as an initial exploratory assessment. The 400-m buffer was used as an approximation of, but did not replicate, the Walk Score methodology, which defines a 5-min walk (approximately 0.25 miles) as the upper threshold for walkable access [18]. The 2-km buffer corresponded to a 5-min drive, calculated using the average speed on general roads in Aichi Prefecture [19]. To assess the robustness of the choice of catchment size, we conducted sensitivity analyses by varying the buffer radius from 300 m to 3 km.

Population and administrative boundary data were obtained at the small-area level (e.g., chō-chō-aza units, which are traditional submunicipal divisions commonly used in Japanese census and administrative records, providing a finer spatial resolution than cities, wards, or towns), based on the 2020 Population Census of Japan. Because these units represent the smallest spatial divisions that remain consistent with policy-relevant administrative boundaries, they were adopted as the unit of analysis. They allow meaningful regional comparisons and detailed spatial analysis. Statistical and boundary data were retrieved from the GIS database of e-Stat, Portal Site of Official Statistics of Japan [16]. Spatial analyses were conducted using ArcGIS Pro (Version 3.4.0; Esri, Redlands, CA, USA)

In Japan, healthcare regions (iryōken) are administrative units designated for planning and managing the development and provision of medical resources within each region [13,20]. These regions are classified into three levels—primary, secondary, and tertiary healthcare regions—each defined by its scope of care and functional role. The primary healthcare region provides routine and general medical services and is typically defined at the municipal level (i.e., cities, towns, or villages). The secondary healthcare region covers a broader area that includes multiple municipalities and delivers inpatient and specialized medical care that can generally be completed within the region. The tertiary healthcare region encompasses the entire prefecture and is responsible for highly specialized and advanced medical services, including those requiring sophisticated technology and expertise.

In Aichi Prefecture and its neighboring prefectures, buffer zones with radii of 300 m, 400 m, 500 m, 1 km, 2 km, and 3 km were generated around medical institutions. These buffers were overlaid on small-area datasets for each secondary healthcare region, which were constructed by linking administrative boundaries and population data. To estimate the population and elderly population within each buffer, this study applied areal weighting. Areal weighting allocates data proportionally based on the spatial overlap between polygons. We estimated the population within each buffer by multiplying the total population of small areas by their respective proportion intersected by the buffer. The population coverage rate was defined as the proportion of the total population in the buffer zones within each secondary healthcare region. The elderly population coverage rate was defined in the same way, but specifically for individuals aged 65 years and older. Both indicators were adopted to assess accessibility. In addition to the walking- and driving-distance assumptions for medical institutions, we evaluated proximity to transit stops and stations. Buffer zones with a radius of 400 m were generated around bus stops and train stations to calculate population and elderly population coverage rates for transit-node proximity.

**Fig 2:**
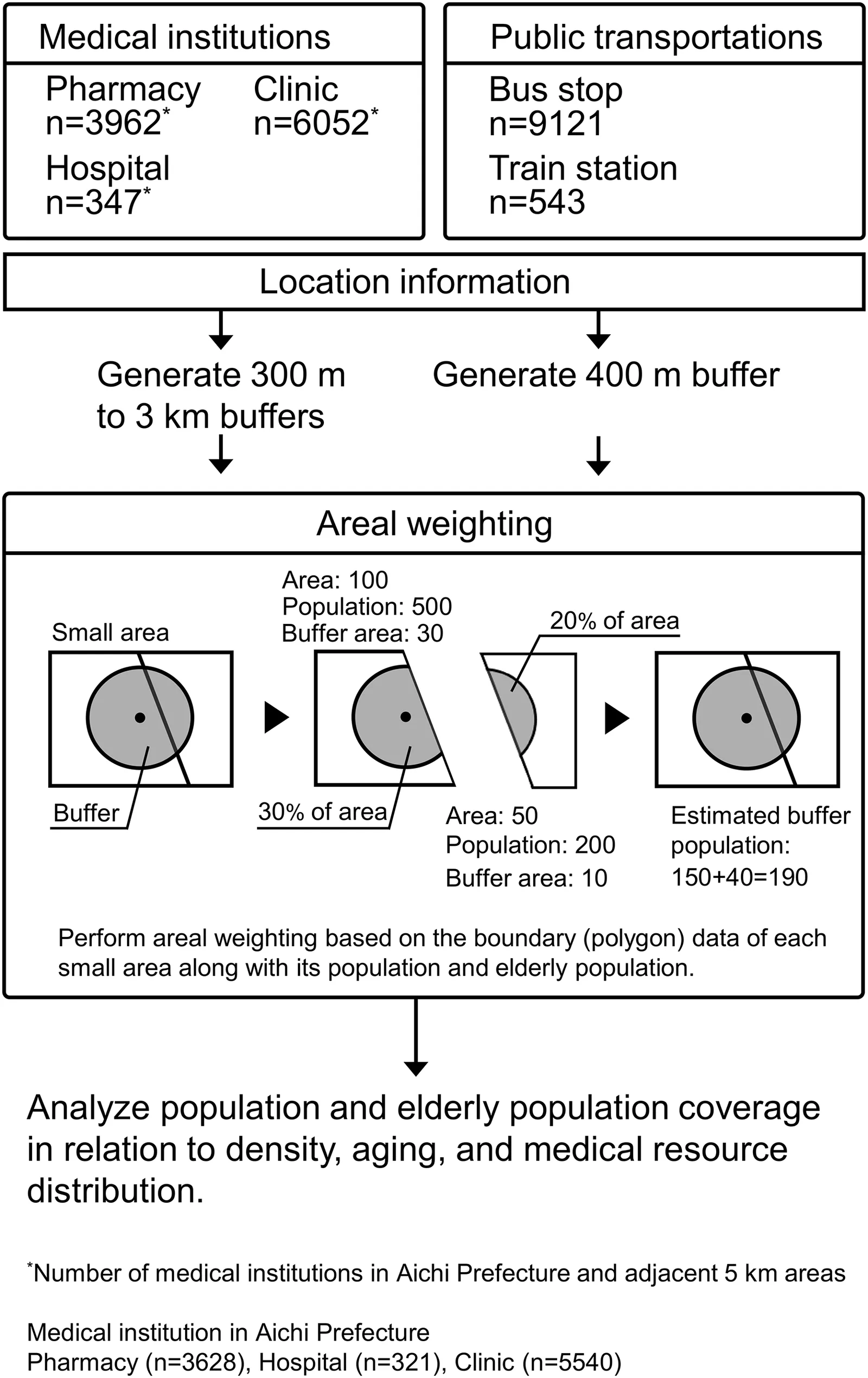
Methods for analyzing population and elderly coverage in relation to density, aging, and medical resource distribution.

We conducted a comprehensive analysis of accessibility to medical institutions in each region based on indicators such as population coverage rates, population density, aging rates (percentage of people aged 65 and over), and the distribution of medical resources (Fig 2).

### Ethics statement

This study used only publicly available, anonymized, aggregate data from the 2020 Population Census of Japan (via e-Stat [16]) and geospatial datasets from the National Land Numerical Information download service of the MLIT [15]. No human participants were contacted or surveyed, and no identifiable personal information was collected or analyzed. Accordingly, the study did not constitute “medical and biological research involving human subjects” as defined by Japan’s Ethical Guidelines for Medical and Biological Research Involving Human Subjects [21], and institutional ethical review was not required.

## Results

**Fig 3:**
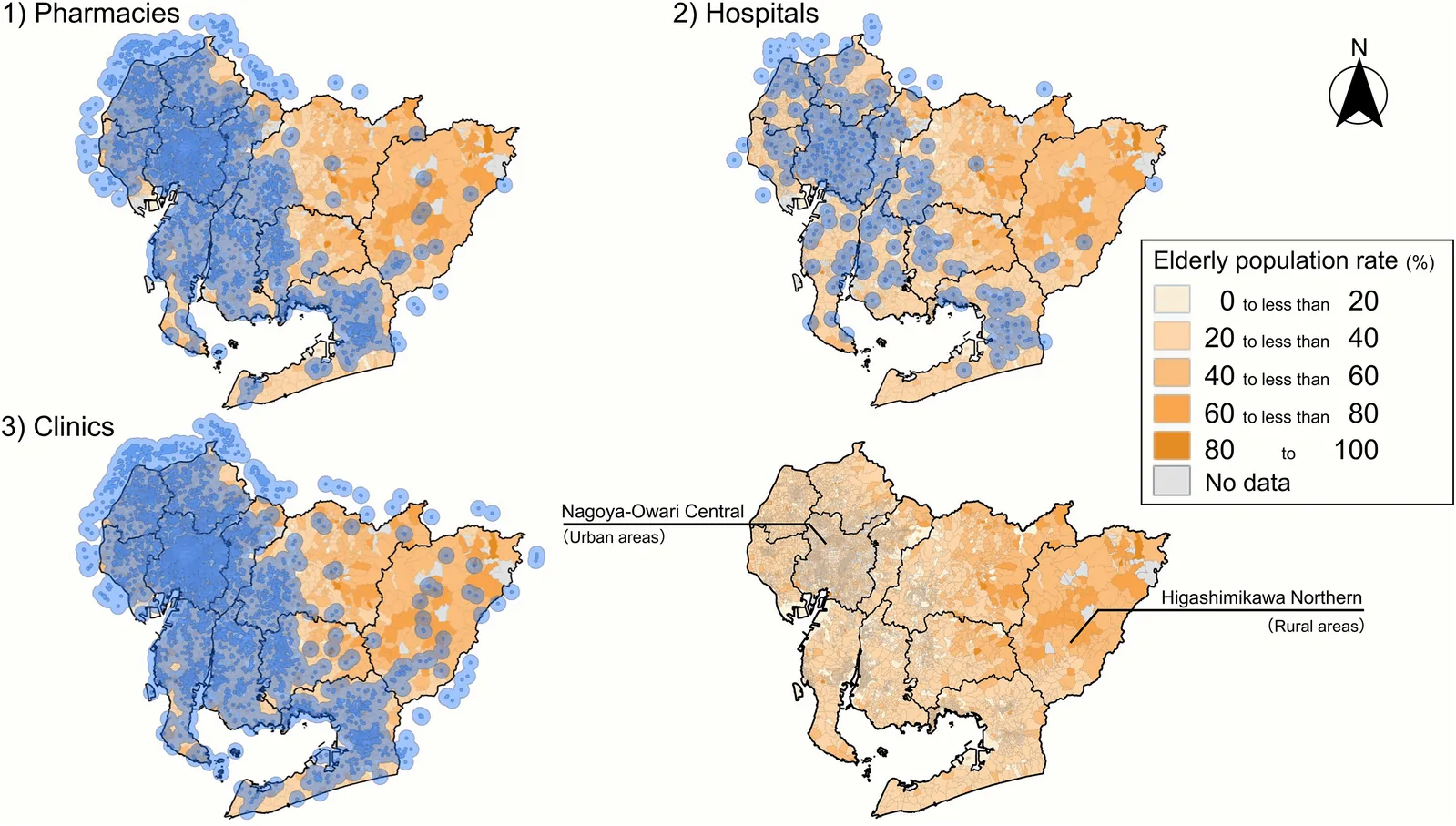
Buffers (400-m and 2-km) around 1) pharmacies, 2) hospitals, and 3) clinics and distribution of the elderly population rate. Created in ArcGIS Pro using publicly available data from e-Stat, the National Land Numerical Information Download Site, and the Regional Bureau of Health and Welfare. The source datasets are licensed under CC BY 4.0 or the Public Data License (Version 1.0) (PDL 1.0) (or an equivalent open-data license). The authors processed and visualized the data to produce this figure.

Fig 3 presents the results of overlaying 400-m and 2-km buffer zones—created around each pharmacy (n = 3,962), hospital (n = 347), and clinic (n = 6,052) in Aichi Prefecture and within 5 km of its prefectural boundary—with the aging rate at the small-area level in Aichi Prefecture. Of these institutions, those located within Aichi Prefecture itself numbered 3,628 pharmacies, 321 hospitals, and 5,540 clinics during the study period. All types of medical institutions were heavily concentrated in the western part of the prefecture, particularly within the Nagoya–Owari Central healthcare region and parts of the Higashimikawa Southern region. In contrast, the northeastern areas had fewer medical institutions.

Regions with aging rates exceeding 60% had few or no medical institutions. Comparison with Fig 1 demonstrated that medical institutions clustered in densely populated lowland areas, whereas mountainous regions remained underserved.

**Fig 4:**
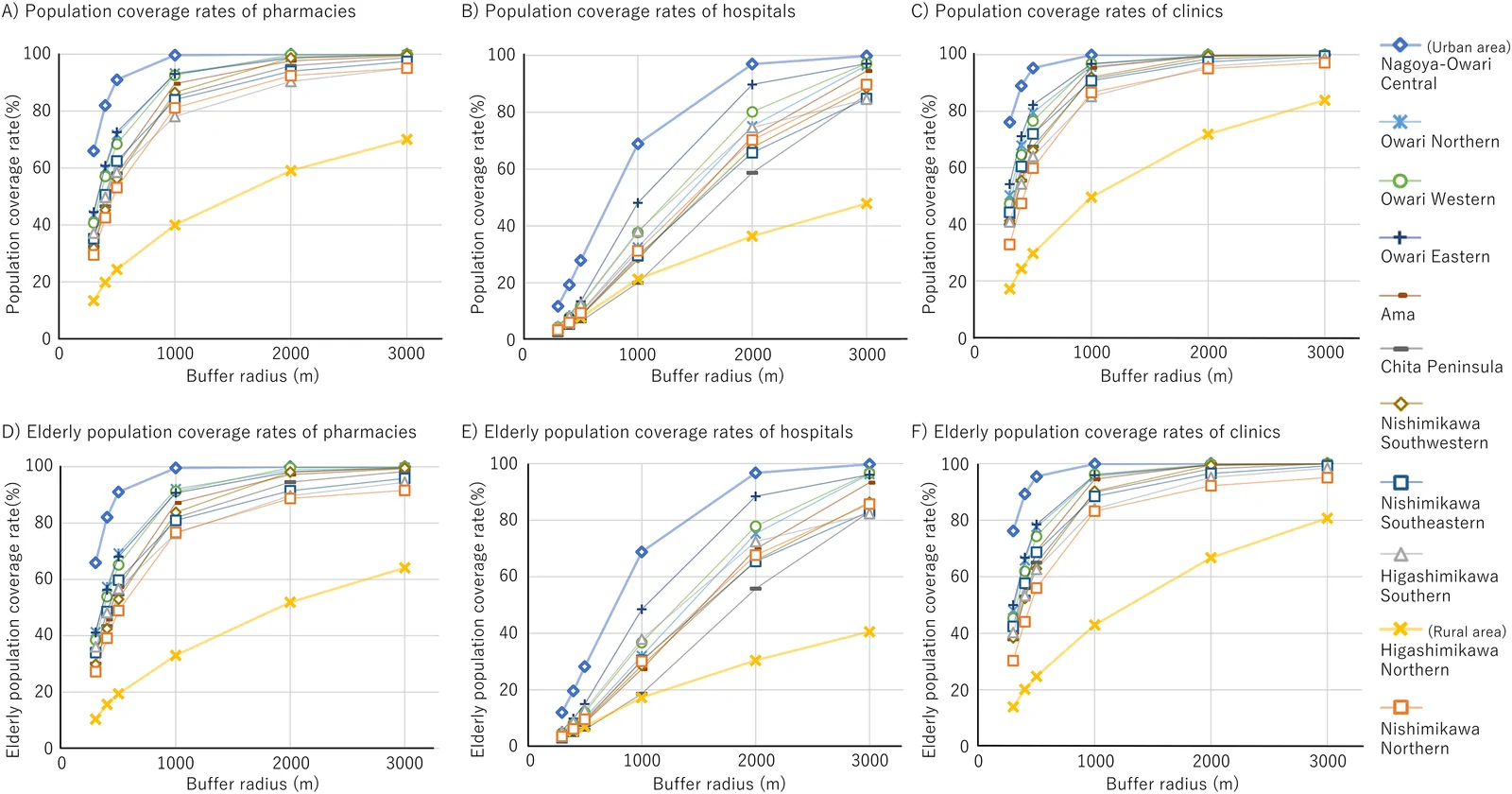
Population coverage rates and elderly population coverage by facility type and secondary healthcare region.

Population coverage rates for each healthcare region are summarized in Fig 4 and S1 Table. In the Nagoya–Owari Central healthcare region—the most populous region (hereafter referred to as the “urban area”)—the coverage rates ranked highest across all types of medical institutions. For the 2-km buffer, the coverage rate nearly reached 100% for all facility types (pharmacy 400 m: 82%; pharmacy 2 km: 100%; hospital 400 m: 19%; hospital 2 km: 97%; clinic 400 m: 89%; clinic 2 km: 100%). In contrast, the Higashimikawa Northern healthcare region—the least populous region (hereafter referred to as the “rural area”)—showed the lowest coverage for pharmacies and clinics, with differences of more than 15 percentage points compared with other regions. Hospital coverage was also lower; under the 2-km buffer (and similarly under the 3-km buffer), coverage remained more than 20 percentage points below that of other regions (pharmacy 400 m: 20%; pharmacy 2 km: 59%; hospital 400 m: 6%; hospital 2 km: 36%; clinic 400 m: 24%; clinic 2 km: 72%).

Excluding the urban and rural areas described above, population coverage rates in the remaining nine healthcare regions were examined by facility type. For pharmacies, coverage rates ranged from 43% to 61% within the 400-m buffer and from 90% to 100% within the 2-km buffer. For hospitals, coverage rates were between 4% and 9% at 400 m and between 59% and 90% at 2 km. For clinics, coverage rates ranged from 47% to 71% at 400 m and from 95% to 100% at 2 km. These findings indicated that even in areas with relatively low coverage within 400 m, accessibility improved substantially when the catchment area extended to 2 km. Pharmacy and clinic coverage exceeded 50% at a 500-m buffer, whereas hospital coverage did not exceed 50% until the buffer was expanded to 2 km.

**Fig 5:**
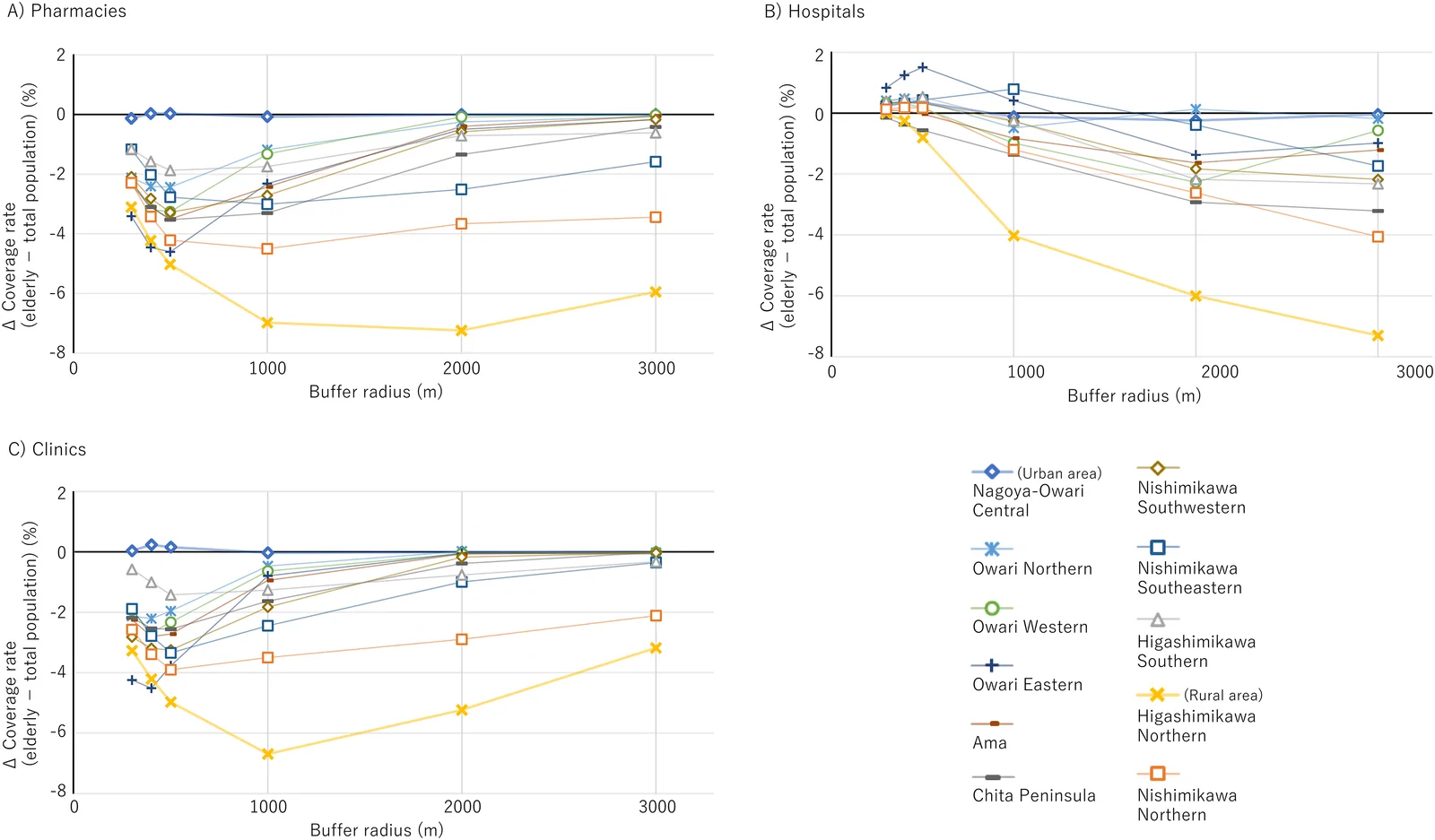
Elderly population coverage rates and differences relative to total population coverage rates (elderly minus total).

Fig 5 shows the differences between elderly population coverage rates and total population coverage rates. Elderly Population coverage rates for each healthcare region are summarized in S2 Table. In the urban area, the two indicators differed only marginally. In contrast, in the rural area, the elderly population coverage rate was consistently lower than the total population coverage rate across all facility types. Specifically, within the rural area, the elderly population coverage rate within 2-km buffer for pharmacies, 3-km buffer for hospitals, and 1-km buffer for clinics was approximately 7% lower than the corresponding total population coverage rate. Most regions exhibited negative differences; however, exceptions were observed for hospitals in several regions and for clinics in the urban area.

Table 3 shows the population and elderly population coverage rates within 400 m of bus stops and train stations. In the urban area, both indicators reached the highest values, at 96%. Conversely, the rural area exhibited the lowest values, with a population coverage rate of 50% and an elderly population coverage rate of 45%.

**Table 3:** Population and elderly population coverage rates within 400 m of bus stops and train stations.

Secondary healthcare region	Total	Elderly
Nagoya-Owari Central	96	96
Owari Northern	81	80
Owari Western	68	69
Owari Eastern	93	92
Ama	65	60
Chita Peninsula	74	71
Nishimikawa Southwestern	72	70
Nishimikawa Southeastern	66	65
Higashimikawa Southern	69	70
Higashimikawa Northern	50	45
Nishimikawa Northern	60	57

Unit: %.

S3 Table presents the correlations between population coverage rates and population density, as well as between population coverage rates and the aging rate. In the full dataset of 11 secondary healthcare regions, positive correlations with R^2^ values greater than 0.70 were observed between population coverage rates and population density for pharmacies and clinics at 300 m and 400 m, and for hospitals at 300 m, 400 m, 500 m, and 1 km. Negative correlations with R^2^ values greater than 0.70 were also observed between population coverage rates and the aging rate for pharmacies and clinics at 2 km and 3 km. However, because these regression analyses were based on only 11 observations, the results may be sensitive to extreme regions. Therefore, we conducted a sensitivity analysis excluding Nagoya–Owari Central and Higashimikawa Northern, which represented the highest-density urban region and the lowest-density rural region, respectively (S4 Table). After excluding these two regions, the previously observed correlations were attenuated, and the correlations between population coverage rates and the aging rate fell below an R^2^ of 0.70 across all facility types and buffer distances. These findings indicate that the regression analyses should be interpreted as descriptive explorations of regional trends rather than inferential evidence of consistent associations.

## Discussion

In urban areas, both the population coverage rate and elderly population coverage rate were high for all types of medical institutions—pharmacies, hospitals, and clinics—indicating a concentrated allocation of healthcare resources. In contrast, in rural areas, the lowest population coverage rates were observed for hospitals at buffer distances greater than 1 km and for pharmacies and clinics across all buffer distances, revealing clear disparities compared with other regions. These results demonstrate the existence of healthcare inequalities between urban and rural areas.

A comparison by buffer distance revealed that, for pharmacies and clinics, regional disparities were more pronounced within the 400-m walking buffer but became less distinct within the 2-km driving buffer. Excluding the rural area, coverage within 2 km exceeded 90% for both facility types. This pattern suggests that private car access may complement geographic accessibility to medical services by improving geographic reachability, particularly in areas with limited spatial proximity to medical facilities in rural areas, consistent with the fact that Aichi Prefecture ranks among the highest in Japan for car ownership and the high proportion of residents who use private vehicles for outpatient visits [22]. However, given the decline in driving ability with age, it is reasonable to question whether reliance on private cars can be sustained as a long-term assumption for ensuring equitable access. In the case of hospitals, the regional differences in coverage rates were relatively small within the walking buffer. This pattern also reflects the regulatory context: hospitals are developed under the criteria of the Medical Care Act and hospital bed functions are strategically planned within secondary healthcare regions under the framework of the regional healthcare vision [20].

In the analysis focusing on the elderly population, the elderly population coverage rate in rural areas was up to approximately 7% lower than the total population coverage rate. Elderly individuals, who typically have higher medical needs, may experience insufficient access to healthcare resources. To address these disparities, strategic reallocation and functional enhancement of medical facilities while considering the spatial distribution of the elderly population in each region are required. Drawing on evidence from comparable rural healthcare settings and the broader literature, complementary measures may help mitigate geographic barriers to healthcare access in rural and mountainous areas with a high concentration of elderly residents. Such measures may include mobile clinics, publicly supported mobile health services, transportation subsidies, and diverse community-based transportation systems. These approaches may complement existing medical institutions and help create an environment in which elderly residents can more readily access necessary medical care within their living areas.

Regarding proximity to transit stops and stations, coverage rates within 400 m of bus stops and train stations were high in urban areas and low in rural areas. This pattern indicates that in rural regions, some areas lack walkable access to healthcare facilities via public transit, highlighting the vulnerability of elderly residents in terms of mobility and the resulting impact on healthcare access. In Aichi Prefecture, the most frequently used mode of transportation for both the general population and older adults is self-driving by private car; the next most common mode is rail use among the general population and walking among older adults [23]. Approximately 25% of older adults do not hold a driver’s license [23], suggesting that the availability of public transportation is an important determinant of healthcare access, particularly for those with limited mobility options. Modal choice also varies substantially by region: in Nagoya City, 36.2% of respondents reported that they “often choose” or “almost always choose” public transportation, whereas this proportion was only 6% in Higashimikawa Northern [22]. This regional gap underscores the potential vulnerability of rural older adults who may face compounded barriers due to limited car access and insufficient transit services. Moreover, UN Sustainable Development Goal Target 11.2 emphasizes the importance of ensuring access to safe, affordable, accessible, and sustainable transport systems—particularly through the expansion of public transportation—with special consideration for vulnerable groups, including older persons [24]. In this context, developing sustainable transportation systems in rural areas experiencing rapid population aging is likely to be critical for maintaining equitable access to healthcare.

In S3 Table, positive correlations (R^2^ > 0.70) between population coverage rates and population density and negative correlations (R^2^ > 0.70) between population coverage rates and the aging rate were observed in the full dataset. However, due to the small sample size (n = 11), much of the observed variation may have been influenced by two extreme regions. In S4 Table, which excludes Nagoya–Owari Central and Higashimikawa Northern, the correlations observed in the full dataset were attenuated. Therefore, the relationships between population coverage rates and population density, as well as between population coverage rates and the aging rate, should be interpreted with caution. In this study, these regression analyses are better viewed as descriptive explorations of regional trends rather than inferential tests providing strong evidence of causal or consistent relationships across regions. Overall, while a tendency toward higher population coverage rates with increasing population density was observed, there was no evidence supporting a simple and consistent association between the aging rate and population coverage rates across the secondary healthcare regions.

Spatial disparities in healthcare access varied depending on residential location, aligning with the result of previous studies [3–9] that have examined the geographic maldistribution of healthcare resources. Furthermore, a distinctive feature of this study is the use of the secondary healthcare region—the fundamental unit of Japan’s regional healthcare planning—as the analytical framework. Under the Medical Care Act and the framework of the regional healthcare vision, the secondary healthcare region serves as a planning unit for hospital bed allocation and the distribution of healthcare resources. By using secondary healthcare regions as the analytical framework, our study clarifies the need for region-specific public interventions to improve healthcare access. The results underscore the policy relevance of targeted measures, including the deployment of telemedicine, integration of mobile health services, expansion of rural transportation schemes, and adaptive spatial planning tailored to aging settlement structures. Specifically, increasing the frequency and coverage of public transportation, introducing alternative mobility options, and providing public support for medical institutions in rural areas are warranted.

Geographic and demographic perspectives can be systematically integrated into healthcare policies to support community care and sustainable allocation of medical resources for elderly individuals. This integration should focus on improving access in underserved rural and mountainous areas and promoting multimodal transportation systems along with healthcare delivery methods suited to local needs. In addition, regional healthcare planning should use specific spatial analysis measures, such as population and elderly population coverage rates, to design targeted strategies that address the unique demographic features and mobility limitations of each area.

Because establishing new medical institutions may be impractical in sparsely populated areas, complementary approaches are likely essential to maintain access. These may include home-based medical care, the use of mobile pharmacy services, and, for time-critical emergencies, public support for rapid transport options, such as physician-staffed helicopter services [25,26]. Collectively, such interventions could help strengthen the local care environment and mitigate access inequities in rural and mountainous regions.

Building on these findings, the analytical approach used in this study may be applied to other regions to examine whether similar spatial patterns of proximity-based potential access to medical institutions are observed. Future studies should expand the scope of analysis to the national level, enabling more comprehensive and region-specific evaluations of healthcare accessibility.

This study had several limitations. First, the buffer distances used in this study were based on general assumptions about walking and driving distances and did not reflect actual travel time or road conditions. As noted by Boscoe et al. [27], areas that appear to fall within a buffer when measured by straight-line distance sometimes lie outside the catchment area when assessed using road networks, potentially leading to underestimation of actual travel times. Furthermore, the 400-m walking buffer adopted in this study approximates, but does not replicate, the Walk Score methodology [18], which evaluates a distance of 400 m (approximately 0.25 miles, or a 5-minute walk) as a complete walkable distance and applies a distance-decay function. However, whereas Walk Score calculates distances based on the actual pedestrian street network, this study used Euclidean (straight-line) distance. These two distance measurement methods are not interchangeable, and the ratio of network distance to Euclidean distance (circuity factor) is generally greater than 1.0; this ratio may be even larger in mountainous terrain. Therefore, the 400-m Euclidean distance buffer applied in this study likely overestimates the actual walkable range implied by the Walk Score standard, particularly in rural areas where access disparities are substantial. Second, our estimates may be subject to over- or under-estimation due to variation in the size and shape of small-area units. Areal weighting assumes that the population is uniformly distributed within each source polygon (in this study, chō-chō-aza units). Consequently, in large and spatially heterogeneous polygons, coverage may be overestimated if residences are concentrated farther from facilities, whereas coverage may be underestimated if residences are concentrated near facilities. Third, this study did not account for functional differences among medical institutions, such as the availability of specific medical care, and could not determine whether available services met actual medical needs. Fourth, the study assessed spatial proximity as the sole indicator of accessibility and did not incorporate non-geographic factors such as waiting time or cost. Additionally, the analysis of proximity to public transit nodes measured only the share of the population living within 400 m of a bus stop or train station, representing nearness to transit nodes rather than actual access to healthcare via transit. This means that residents counted as “covered” may not have functional transit routes connecting them to pharmacies, hospitals, or clinics. In rural regions, where services are sparse and infrequent, this 400 m proximity metric likely overestimates the true accessibility of healthcare through public transportation. Therefore, the transit coverage figures should be interpreted cautiously, as they reflect proximity to transit nodes and do not necessarily correspond to effective healthcare reachability by public transit. Fifth, we used the most recent datasets available, which we considered to best reflect current conditions. However, the reference years differed across data sources (e.g., the 2020 Population Census, 2022 bus stop data, 2023 railway data, and a 2025 pharmacy list). This mismatch may introduce time-lag bias with respect to actual present-day accessibility, because changes occurring between these years—such as population shifts, opening or closure of facilities, relocation of bus stops, and the opening of new railway segments—could cause our estimates to deviate systematically from real-world conditions. Moreover, this temporal mismatch is directionally asymmetric: hospital and clinic datasets correspond to 2020, whereas pharmacy data reflect the situation in 2025. Support for the reorganization of hospital bed functions has been promoted toward the realization of the regional healthcare vision. In 436 hospitals subject to re‑evaluation, the number of acute care beds is projected to decrease from 40,300 in 2017–29,100 in 2025, representing an estimated reduction of approximately 28% [28]. Concurrently, financial pressures have precipitated the closure of smaller public hospitals, particularly in low-density rural areas. The number of hospital beds was 1.246 million in 2018, but is estimated to decrease to 1.218 million by 2025 [29]. Consequently, the actual current hospital coverage rates in these regions may be lower than those estimated in this study.

Nevertheless, we considered these datasets to represent the best practicably obtainable data in Japan at present, and all analyses were conducted using this compiled dataset. Finally, external validity (transportability) is not guaranteed and should be interpreted with caution because (i) spatial structure, transport networks, age demographics, and healthcare service arrangements vary across prefectures; (ii) the indicators used in this study (Euclidean distance, fixed buffers, and no adjustment for service capacity) may perform differently in metropolitan areas than in remote rural settings; and (iii) time lags between data layers are not uniform.

Future research should incorporate network-based analyses that evaluate actual travel time using road networks, assess accessibility according to medical function and specialty, and include multidimensional factors such as elderly individuals’ transportation options and mobility capabilities. Moreover, evaluating the effectiveness of complementary measures—such as local transportation support services or mobile clinics—will be essential in developing inclusive healthcare access strategies. Future studies should also incorporate interviews and surveys with residents, including elderly individuals, in the 11 secondary healthcare regions to capture diverse needs and preferences in relation to healthcare access and related challenges. Integrating the perspectives of all community members will provide comprehensive insights into obstacles experienced in daily life and support the development of effective, user-centered policies aimed at improving healthcare accessibility and overall well-being across various geographical settings.

## Conclusion

This study quantitatively evaluated geographic accessibility to medical institutions in Aichi Prefecture using GIS-based analysis, incorporating catchment areas representing walking and driving distances. The findings revealed a concentration of medical institutions in urban areas and lower coverage rates in rural areas. The Higashimikawa Northern region recorded the lowest values within the 2-km buffer across all facility types: pharmacies (59%), hospitals (36%), and clinics (72%), underscoring pronounced regional disparities in healthcare accessibility. Spatial disparities in proximity to medical institutions were more pronounced among the elderly population, who experienced coverage rates approximately 7% lower than the total population in rural areas, highlighting a persistent accessibility gap for this high-need demographic. Urban areas also demonstrated high proximity to transit stops and stations, with 96% of residents living within 400 m of bus stops or train stations, whereas this proportion fell below 50% in rural areas. These findings suggest that rural residents, particularly older adults, may face limited proximity to nearby transit stops and stations, although this indicator does not directly measure effective healthcare access by public transportation. Although a 100% coverage rate in urban areas indicates high geographic proximity and good potential access, it does not necessarily translate into ease of obtaining care in practice, as long waiting times and other service constraints may still occur. These results highlight the existence of healthcare access inequalities shaped by residential location and available modes of transportation.

The outcomes of this study provided valuable evidence to guide regional healthcare policy planning and the optimal allocation of medical resources. In particular, the findings are expected to contribute to identifying key challenges and formulating strategies for advancing community-based integrated care and building sustainable healthcare systems, especially in areas affected by population aging and decline.

## Supporting information

S1 Table Population coverage rates. (XLSX)

S2 Table Elderly population coverage rates. (XLSX)

S3 Table Correlations between population coverage rates and population density and between population coverage rates and aging rates (all regions of Aichi Prefecture). (XLSX)

S4 Table Correlations between population coverage rates and population density and between population coverage rates and aging rates (excluding regions: Higashimikawa Northern (lowest population, rural) and Nagoya-Owari Central (highest population, urban)). (XLSX)
