# Supplementary material for: Accessibility to medical institutions in Aichi Prefecture: A geographic information systems-based analysis
Source: PLoS One. 2026 Aug 26;21(8):e0356748. doi: 10.1371/journal.pone.0356748 (PMC13514043; doi:10.1371/journal.pone.0356748)
Supplement: Supplementary file 1 [file pone.0356748.s006.docx]

Response to Reviewer 3

We wish to express our sincere gratitude for your thorough and meticulous review of our manuscript. Your expert assessment and well-considered remarks have been instrumental in enhancing the scholarly rigor and overall quality of our work. Each of your comments has been examined with the utmost care, and corresponding revisions have been incorporated into the manuscript where appropriate.

The following presents a detailed, point-by-point response, with page and line numbers provided to indicate the precise locations of the modifications in the revised version.

**1. One key point relates to the theoretical positioning of the study. The introduction references relevant prior work; however, it does not yet fully engage with more recent international literature examining health accessibility inequalities in aging contexts. Over the past five years, this topic has received growing attention, particularly in Western countries where older populations tend to remain in geographically isolated regions as younger populations consolidate in urbanized centers. For example, in the study "Travel-time accessibility and adaptive spatial planning solutions for the healthcare system" (npj Health Systems) analyze similar accessibility gaps and propose adaptive planning strategies in response to demographic aging. Likewise, in the study "Mapping population dynamics at local scales using spatial networks"(Complexity) the authors document emerging dual settlement patterns—urban concentration versus rural aging—which closely parallel the dynamics observed in Aichi Prefecture. Positioning the manuscript more explicitly within this evolving global discussion would significantly enhance its relevance.**

Thank you for your insightful comment. As you correctly pointed out, our literature review did not sufficiently incorporate recent international studies and prior work on geographic information systems. In response, we have added new citations and revised the Literature Review section accordingly:

“In recent years, populations have increasingly concentrated in urban areas, whereas rural regions have experienced rapid population aging [1].” (page 3, lines 50–52)

“Moreover, Balsa-Barreiro et al. [9] proposed strategic approaches to hospital location planning to better respond to population aging.” (page 4, lines 70–71)

We also added four new references (4, 5, 7 and 8) to the literature review (page 4, lines 61, 67 and 69), in addition to references [1] and [9] cited above.

New references:

1. Balsa-Barreiro J, Morales AJ, Lois-González RC. Mapping Population Dynamics at Local Scales Using Spatial Networks. Complexity. 2021, 8632086. doi: 10.1155/2021/8632086

4. Krasniuk S, Crizzle AM. Impact of health and transportation on accessing healthcare in older adults living in rural regions. Transp Res Interdiscip Perspect. 2023;21:100882.

5. Asante D, McLachlan CS, Pickles D, Isaac V. Understanding Unmet Care Needs of Rural Older Adults with Chronic Health Conditions: A Qualitative Study. Int J Environ Res Public Health. 2023;20(4):3298. doi:10.3390/ijerph20043298.

7. da Silva CN, Rocha TAH, Amaral PV, Elahi C, Thumé E, Thomaz EBAF, et al. Comprehending the lack of access to maternal and neonatal emergency care: Designing solutions based on a space-time approach. PLoS One. 2020; 15(7): e0235954. doi: 10.1371/journal.pone.0235954.

8. Kajimoto S, Suzuki S, Okada H. Analysis of pharmacy accessibility and function in Wakayama Prefecture using geographic information system. Yakugaku Zasshi. 2025;145(7):639-643. doi:10.1248/yakushi.25-00008.

9. Balsa-Barreiro J, Batista SFA, Hannoun GJ, Menendez M. Travel-time accessibility and adaptive spatial planning solutions for the healthcare system. npj Health Systems. 2025;2:24. doi:10.1038/s44401-025-00028-1.

**2. Methodologically, although the use of straight-line buffers (400 m and 2 km) is understandable for an initial exploratory assessment, the rationale behind selecting these specific thresholds would benefit from deeper justification. Recent research suggests that accessibility thresholds are most meaningful when connected to travel-time criteria rather than linear distance. For instance, In the previos study the authors propose a 30-minute travel-time threshold as a critical standard, aligned with prior work in spatial accessibility assessment across multiple health systems. Connecting the selected distances to either mobility behavior, national planning benchmarks, or established international standards would strengthen methodological clarity and comparability.**

Thank you for your comment. As you noted, the 400 m and 2 km straight-line buffer distances were used for an initial exploratory assessment, so we have noted this in method (page 10, lines 164–165). While implementing travel-time thresholds would be methodologically desirable, the necessary data for accurate network-based travel-time calculations were not available at the time of this study.

To demonstrate the robustness of our indicator, we generated buffers of 300/400/500 m and 1/2/3 km, calculated population coverage rates and elderly population coverage rates, and visualized them accordingly. We incorporated these findings into newly added Fig 4 (page 14, lines 253–254), Fig 5 (page 15, line 275–page 16, line 276), S1 Table (page 28, line 514), S2 Table (page 28, line 515)

We also added the following clarifications in the Discussion to acknowledge this methodological limitation and propose future directions:

“We tested how results changed when buffer distance varied from 300 m to 3 km.” (page 2, lines 27–28)

“To assess the robustness of the choice of catchment size, we conducted sensitivity analyses by varying the buffer radius from 300 m to 3 km.” (page 10, lines 168–169)

“In Aichi Prefecture and its neighboring prefectures, buffer zones with radii of 300 m, 400 m, 500 m, 1 km, 2 km, and 3 km were generated around medical institutions.” (page 11, lines 189–190)

“First, the buffer distances used in this study were based on general assumptions about walking and driving distances and did not reflect actual travel time or road conditions. As noted by Boscoe et al. [27], areas that appear to fall within a buffer when measured by straight-line distance sometimes lie outside the catchment area when assessed using road networks, potentially leading to underestimation of actual travel times.” (page 21, lines 375–379)

“Future research should incorporate network-based analyses that evaluate actual travel time using road networks, assess accessibility according to medical function and specialty, and include multidimensional factors such as elderly individuals’ transportation options and mobility capabilities.” (page 23, line 402– line 404)

**3. The discussion section could also be further developed. As the authors note, Aichi Prefecture has the highest rate of private vehicle ownership in Japan, which may temporarily compensate for reduced spatial accessibility in rural areas. However, this finding invites deeper interpretation: given the elderly population’s declining driving capacity, it is reasonable to question whether car dependency remains a sustainable long-term assumption. The observed discrepancy in Table 4—where elderly coverage in rural areas is consistently lower—makes this issue particularly relevant.**

Thank you for highlighting this important point. As you noted, the sustainability of car dependency among the elderly population warrants further discussion. We have expanded the Discussion section to include the following:

“This pattern suggests that private car access plays a complementary role in securing medical services in rural areas, consistent with the fact that Aichi Prefecture ranks among the highest in Japan for car ownership and the high proportion of residents who use private vehicles for outpatient visits [22]. Such factors may partially offset reduced spatial accessibility in less densely populated areas. However, given the decline in driving ability with age, it is reasonable to question whether reliance on private cars can be sustained as a long-term assumption for ensuring equitable access.” (page 18, lines 307–313)

“In Aichi Prefecture, the most frequently used mode of transportation for both the general population and older adults is self-driving by private car; the next most common mode is rail use among the general population and walking among older adults [23]. Approximately 25% of older adults do not hold a driver’s license [23], suggesting that the availability of public transportation is an important determinant of healthcare access, particularly for those with limited mobility options. Modal choice also varies substantially by region: in Nagoya City, 36.2% of respondents reported that they “often choose” or “almost always choose” public transportation, whereas this proportion was only 6% in Higashimikawa Northern [22]. This regional gap underscores the potential vulnerability of rural older adults who may face compounded barriers due to limited car access and insufficient transit services.” (page 19, lines 327–336)

New references:

22. Aichi Prefecture Government. Aichi Prefecture regional public transportation plan. 2024. Available from: https://www.pref.aichi.jp/uploaded/attachment/520008.pdf  [cited 2025 Feb 10].

23. Aichi Prefecture Government. Survey results on traffic safety measures for the elderly. 2019. Available from: https://www.pref.aichi.jp/police/koutsu/koureisha/gd/documents/siryou3.pdf  [cited 2025 Feb 10].

**4. In addition, the policy implications, though mentioned, could be made more concrete. The results strongly support the relevance of targeted interventions such as telemedicine deployment, integration of mobile health services, expansion of rural transportation schemes, and adaptive spatial planning approaches tailored to aging settlement structures.**

Thank you for this suggestion. We have expanded our discussion of policy implications to include feasible interventions for rural areas, such as home-based medical care, mobile pharmacy services, and physician-staffed emergency transport:

“The results underscore the policy relevance of targeted measures, including the deployment of telemedicine, integration of mobile health services, expansion of rural transportation schemes, and adaptive spatial planning tailored to aging settlement structures. Specifically, increasing the frequency and coverage of public transportation, introducing alternative mobility options, and providing public support for medical institutions in rural areas are warranted.

Because establishing new medical institutions may be impractical in sparsely populated areas, complementary approaches are likely essential to maintain access. These may include home-based medical care, the use of mobile pharmacy services, and, for time-critical emergencies, public support for rapid transport options, such as physician-staffed helicopter services [25,26]. Collectively, such interventions could help strengthen the local care environment and mitigate access inequities in rural and mountainous regions.” (page 20, line 358–page 21, line 369)

New references:

25. Nelson C, Malone M, Williams MM, Smith Fawzi MC, Bennet J, Hill C, et al. Mobile health clinics in the United States. Int J Equity Health. 2020;19:40. doi:10.1186/s12939-020-1135-7.

26. Ackermann A, Pappinen J, Nurmi J, Nordquist H, Saviluoto A, Mannila S, et al. A scenario-based approach to optimizing cost-effectiveness of physician-staffed Helicopter Emergency Medical Services compared to ground-based Emergency Medical Services in Finland. Scand J Trauma Resusc Emerg Med. 2024;32:60. doi:10.1186/s13049-024-01231-z.

**5. Finally, I would like to highlight a technical concern: several figures appear in low resolution and would benefit from being resubmitted with higher visual clarity. Because the maps are central to the paper’s main argument, improving their readability—including clearer legends, labels, and scale indicators—will substantially enhance the manuscript’s accessibility and scientific value.**

Thank you for pointing this out. We agree that the resolution of several figures was insufficient. We will resubmit all figures in higher resolution with clearer legends, labels, and scale indicators to improve readability and enhance the scientific value of the manuscript.
