# Supplementary material for: Accessibility to medical institutions in Aichi Prefecture: A geographic information systems-based analysis
Source: PLoS One. 2026 Aug 26;21(8):e0356748. doi: 10.1371/journal.pone.0356748 (PMC13514043; doi:10.1371/journal.pone.0356748)
Supplement: Supplementary file 2 [file pone.0356748.s007.docx]

Response to Reviewer #1

We wish to express our sincere gratitude for your constructive and insightful comments, which have greatly contributed to improving the clarity, depth, and policy relevance of our manuscript. Each of your suggestions has been carefully considered, and corresponding revisions have been incorporated to strengthen the discussion, provide practical frameworks for integrating geographic and demographic perspectives into healthcare policy, address rural–urban disparities in access to pharmacies, hospitals, and clinics for elderly populations, and highlight directions for future research involving direct engagement with elderly residents. The following presents our detailed, point-by-point responses, with page and line numbers indicating the exact locations of the modifications in the revised version.

1. **Authors need to show in a framework as to how geographic and demographic perspectives can be integrated into healthcare policy to support community care and sustainable resource allocation for senior citizens.**

We sincerely thank you for this valuable comment. We agree that systematically integrating geographic and demographic perspectives into healthcare policy is essential to strengthen community care and ensure sustainable allocation of medical resources for senior citizens. Based on your suggestion, we have added the following explanation to the Discussion section:

“Geographic and demographic perspectives can be systematically integrated into healthcare policies to support community care and sustainable allocation of medical resources for elderly individuals. This integration should focus on improving access in underserved rural and mountainous areas and promoting multimodal transportation systems along with healthcare delivery methods suited to local needs. In addition, regional healthcare planning should use specific spatial analysis measures, such as population and elderly population coverage rates, to design targeted strategies that address the unique demographic features and mobility limitations of each area.” (page 21, lines 374–380)

2. **Discussion needs to be elaborated, and authors need to come up with recommendation and a framework to show how rural-urban disparities in access to three healthcare institutions—pharmacies, hospitals, and clinics—can be improved via better policy and resource allocation for the elderly.**

We sincerely appreciate your insightful suggestion to strengthen the Discussion section by focusing on policy recommendations and a framework to reduce rural–urban disparities in access to pharmacies, hospitals, and clinics for elderly populations. We have added the following explanation to the Discussion section:

“To address these disparities, strategic reallocation and functional enhancement of medical facilities while considering the spatial distribution of the elderly population in each region are required. Specifically, in rural and mountainous areas with a high concentration of elderly residents, the introduction of mobile clinics and publicly supported mobile health services to complement existing medical institutions is expected to be effective. Furthermore, to alleviate mobility constraints among the elderly, it is necessary to strengthen transportation support by enhancing public transportation services and expanding transportation cost subsidy programs, while promoting the development of diverse, community-based transportation modes. These measures can create an environment where elderly individuals can access the necessary medical care within their living areas more readily, thereby reducing geographic disparities in healthcare accessibility between urban and rural regions. In healthcare policy planning, positioning such comprehensive interventions and implementing flexible resource allocation tailored to regional characteristics are of critical importance.” (page 18, line 320–page 19, line 332)

3. **What is the future research direction in terms of asking/interviewing the elderly through interviews and personally administered survey with the elderly population living in this 11 geographical areas, as to what they need to make life /old aged people conformable in context of medical facilities access, availability, and affordability as well as other elderly needs for real policy impact.**

We appreciate your insightful suggestion to incorporate direct input from elderly residents in future research. We have added the following statement to the Discussion section:

“Future studies should also incorporate interviews and surveys with residents, including elderly individuals, in the 11 secondary healthcare regions to capture diverse needs and preferences in relation to healthcare access and related challenges. Integrating the perspectives of all community members will provide comprehensive insights into obstacle experienced in daily life and support the development of effective, user-centered policies aimed at improving healthcare accessibility and overall well-being across various geographical settings.” (page 24, lines 424–429)
