# Supplementary material for: Accessibility to medical institutions in Aichi Prefecture: A geographic information systems-based analysis
Source: PLoS One. 2026 Aug 26;21(8):e0356748. doi: 10.1371/journal.pone.0356748 (PMC13514043; doi:10.1371/journal.pone.0356748)
Supplement: Supplementary file 3 [file pone.0356748.s008.docx]

Response to Reviewer #5

We sincerely thank you for the thorough and constructive comments on our manuscript, including both the positive assessments of novelty and methodological strengths, and the detailed suggestions for improvement.
We have carefully addressed each major and minor concern in this revision, as detailed below.

**Criterion 1–6**

We have confirmed.

**Criterion 7 — Reporting guidelines and data availability
Partially met — minor gaps.
Data availability statement ("All relevant data are within the manuscript and its Supporting Information files") is acceptable given that all source data come from public repositories (e-Stat, MLIT, Tokai-Hokuriku Bureau). However, the geocoded pharmacy point layer (the processed output from the CSV Address Matching Service) is a derived dataset that does not exist in any public repository. Ideally this derived dataset should be deposited. Supporting Tables S1-S3 cover the coverage rates and correlations.**
Regarding the request to deposit the derived dataset (i.e., the geocoded pharmacy point layer produced from the CSV Address Matching Service), we acknowledge that this dataset does not currently exist in any public repository. While we are not making this dataset directly available as a Supplementary Table at this stage, we are willing to provide it upon reasonable request to the corresponding author, in accordance with data-sharing policies. This approach ensures that all source data remain accessible while respecting any potential usage constraints of the derived geocoded layer. We added the Data availability section as follows.

“Data availability

All relevant source data used in this study are publicly available from official repositories: Ministry of Land, Infrastructure, Transport and Tourism (MLIT) datasets [15], e-Stat [16], and the Tokai-Hokuriku Regional Bureau[17]. The minimal data set is fully disclosed in Table 2. Supporting Tables S1-S4, included as supplementary information, provide detailed coverage rates and correlations related to the datasets.

The geocoded pharmacy point layer, which is a derived dataset produced using the CSV Address Matching Service [14], does not currently exist in any public repository. While this derived dataset is not included as a supplementary file, it is available from the corresponding author, M.N., upon reasonable request, subject to data-sharing policies and any usage restrictions.” (page 28, lines 500–509)

**No formal reporting checklist (e.g., STROBE for observational studies) is provided. While STROBE is primarily designed for epidemiological studies, an ecological GIS study using population data falls within its scope; at least, the authors should check and confirm whether any relevant checklist applies to this study type.**
With respect to the reporting checklist, we reviewed available guidelines. We did not identify any checklist that fully matches the scope and methodological characteristics of this ecological GIS study. Although some items in existing checklists are not applicable, we elected to use the STROBE Statement—Checklist of items that should be included in reports of cross-sectional studies as the most relevant framework. This completed checklist has been submitted as an "others" file entitled “for the reviewer #5 STROBE” for editorial assessment.

**Major concerns
M1 — Regression analyses with N=11: insufficient power and outlier sensitivity
The simple regression analyses comparing population coverage rates against population density and aging rate are conducted with N=11 observations (one per secondary healthcare region). At this sample size, the analyses have extremely low statistical power and R² values are easily driven by one or two outliers. The contrast between Higashimikawa Northern (density 50/km², aging rate 38.5%) and Nagoya-Owari Central (density 6,785/km², aging rate 24.2%) is so extreme that these two regions likely account for most of the observed variance.
Required action: The authors should either (a) explicitly acknowledge in the text that the regression results are highly sensitive to outlier regions and should be treated as descriptive rather than inferential, or (b) report the change in R² when the two extreme regions (Higashimikawa Northern and Nagoya-Owari Central) are excluded, to show whether the relationships hold in the mid-range regions. The current text presents R²>0.70 as a "relatively strong positive correlation" without this caveat; this is overconfident given the sample size.**

Thank you for this insightful comment. We fully agree that the regression analysis in our study, with a small sample size (N = 11), is highly sensitive to extreme values and therefore requires cautious interpretation. Accordingly, we have substantially revised the wording in the relevant sections of the manuscript to clearly state that these regressions should be regarded as descriptive explorations rather than inferential analyses. In addition, we conducted a sensitivity analysis excluding the two extreme regions (Higashimikawa Northern and Nagoya-Owari Central), and reported the results in Table S4. The manuscript has been revised as follows.

“S3 Table presents the correlations between population coverage rates and population density, as well as between population coverage rates and the aging rate. In the full dataset of 11 secondary healthcare regions, positive correlations with R² values greater than 0.70 were observed between population coverage rates and population density for pharmacies and clinics at 300 m and 400 m, and for hospitals at 300 m, 400 m, 500 m, and 1 km. Negative correlations with R² values greater than 0.70 were also observed between population coverage rates and the aging rate for pharmacies and clinics at 2 km and 3 km. However, because these regression analyses were based on only 11 observations, the results may be sensitive to extreme regions. Therefore, we conducted a sensitivity analysis excluding Nagoya–Owari Central and Higashimikawa Northern, which represented the highest-density urban region and the lowest-density rural region, respectively (S4 Table). After excluding these two regions, the previously observed correlations were attenuated, and the correlations between population coverage rates and the aging rate fell below an R² of 0.70 across all facility types and buffer distances. These findings indicate that the regression analyses should be interpreted as descriptive explorations of regional trends rather than inferential evidence of consistent associations.” (page 17, line 299–page 18, line 313)

“In S3 Table, positive correlations (R² > 0.70) between population coverage rates and population density and negative correlations (R² > 0.70) between population coverage rates and the aging rate were observed in the full dataset. However, due to the small sample size (n = 11), much of the observed variation may have been influenced by two extreme regions. In S4 Table, which excludes Nagoya–Owari Central and Higashimikawa Northern, the correlations observed in the full dataset were attenuated. Therefore, the relationships between population coverage rates and population density, as well as between population coverage rates and the aging rate, should be interpreted with caution. In this study, these regression analyses are better viewed as descriptive explorations of regional trends rather than inferential tests providing strong evidence of causal or consistent relationships across regions. Overall, while a tendency toward higher population coverage rates with increasing population density was observed, there was no evidence supporting a simple and consistent association between the aging rate and population coverage rates across the secondary healthcare regions.” (page 21, lines 367–379)

“S4 Table. Correlations between population coverage rates and population density and between population coverage rates and aging rates (excluding regions: Higashimikawa Northern (lowest population, rural) and Nagoya-Owari Central (highest population, urban)).” (page 33, lines 603–605)

**M2 — Euclidean buffer vs. Walk Score network distance: methodological inconsistency
The 400m walking buffer is justified by citing the Walk Score methodology (ref 18: this seems a methodology webpage, not a peer-reviewed source), which assigns full walkability credit up to 400 m (0.25 miles, ~5-min walk) before applying a distance-decay function. Walk Score, however, calculates distances using the actual pedestrian street network, not straight-line (Euclidean) distance. The authors acknowledge Euclidean distance as a general spatial limitation elsewhere in the paper (citing Boscoe et al. 2012 for driving accessibility), but they do not address the specific inconsistency that the citation they invoke to justify the 400m threshold, Walk Score, itself relies on network distances. The two approaches are not interchangeable: the detour index (ratio of network to Euclidean distance) consistently exceeds 1.0 in urban street grids and can be substantially higher in mountainous terrain, meaning the Euclidean 400m buffer overstates walkable coverage relative to what the Walk Score standard actually implies, with the effect most pronounced in the rural areas where access gaps are largest.
Required action: The authors should revise the Walk Score citation language to make clear that their Euclidean buffer approximates but does not replicate the Walk Score methodology, and add a sentence acknowledging this specific inconsistency and its directional effect on coverage estimates in mountainous versus urban settings.**

Thank you for pointing out this methodological inconsistency. We agree that Euclidean distance and Walk Score’s network distance calculation are not interchangeable. To address this, we have revised the text to clarify that our 400m Euclidean buffer approximates but does not replicate the Walk Score methodology. We also added a statement noting that Euclidean buffers likely overestimate walkable coverage, particularly in rural and mountainous areas, due to higher detour index values. We modified the method section and added an explanation to the limitation section as follows.

“The 400-m buffer was used as an approximation of, but did not replicate, the Walk Score methodology, which defines a 5-min walk (approximately 0.25 miles) as the upper threshold for walkable access [18].” (page 10, lines 176–178)

“Furthermore, the 400-m walking buffer adopted in this study approximates, but does not replicate, the Walk Score methodology [18], which evaluates a distance of 400 m (approximately 0.25 miles, or a 5-minute walk) as a complete walkable distance and applies a distance-decay function. However, whereas Walk Score calculates distances based on the actual pedestrian street network, this study used Euclidean (straight-line) distance. These two distance measurement methods are not interchangeable, and the ratio of network distance to Euclidean distance (circuity factor) is generally greater than 1.0; this ratio may be even larger in mountainous terrain. Therefore, the 400-m Euclidean distance buffer applied in this study likely overestimates the actual walkable range implied by the Walk Score standard, particularly in rural areas where access disparities are substantial.” (page 23, line 415– page 24, line 424)

**Minor concerns
m1 — Geocoding success rate not reported
Pharmacy locations were derived by geocoding 3,962 addresses (of which 3,628 are in Aichi Prefecture) using the University of Tokyo CSV Address Matching Service. The geocoding success rate (proportion of addresses successfully matched) and the spatial precision of matches are not reported. Failed matches or low-confidence matches would create gaps in the pharmacy coverage map that are invisible in the current analysis.
Required action: Report the geocoding success rate (number/percentage of addresses successfully geocoded) and the precision level of matches (e.g., address-level vs. street-level vs. area-level). If any addresses failed geocoding, state what proportion this represents.**

Thank you for this valuable comment. We agree that geocoding success rate and precision levels are important for interpreting the results. We have now included these details in the methods section:

“All 3,962 pharmacy addresses were successfully geocoded using the CSV Address Matching Service. These addresses were geocoded with varying levels of spatial precision. Among the 3,628 pharmacies located within Aichi Prefecture, 3,250 (approximately 89.7%) were matched to the most detailed geographic units corresponding to city blocks and individual lot numbers. Additionally, 294 addresses were matched to subdivisions roughly equivalent to neighborhood sections (chōme and kōaza), 79 to larger administrative subdivisions similar to wards or local districts (ōaza), 1 to the ward level within designated cities, and 4 to the broader municipality level (city, town, or village). For the entire dataset of 3,962 pharmacies, 3,525 (about 89.0%) were geocoded at the finest level of city block and lot number, 313 at neighborhood subdivision level, 119 at larger district level, 1 at ward level, and 4 at municipality level.” (page 8, line 139–page 9, line 149)

**m2 — Temporal mismatch between data layers is asymmetric
The authors acknowledge the time-lag limitation (p.23, lines 406-413), the study combines 2020 Census population data with 2022 bus stop data, 2023 railway data, and a 2025 pharmacy list. This is reasonable given data availability constraints. However, the discussion does not note that this mismatch is directionally asymmetric: pharmacy data is from 2025 (most current), while hospital/clinic data is from 2020 (MLIT). Between 2020 and 2025, Japan's Regional Healthcare Vision has driven national bed restructuring (targeting a 30% reduction in acute-phase hospital beds), and mounting financial pressures on smaller public hospitals have contributed to closures nationally. The hospital layer (321 hospitals in Aichi Prefecture, 2020) may therefore overstate current availability, particularly in lower-density areas where smaller hospitals are more financially vulnerable.
Required action: Add a sentence to the limitations paragraph explicitly noting that the hospital/clinic data (2020) is 5 years older than the pharmacy data (2025), and that hospital restructuring between these years may mean that current hospital coverage rates in rural areas are lower than reported.**

Thank you for this important observation. We agree that hospital and clinic data (2020) are five years older than pharmacy data (2025), and that restructuring between these years may reduce current hospital availability, especially in rural areas. We have added this statement to the limitations section.

“Moreover, this temporal mismatch is directionally asymmetric: hospital and clinic datasets correspond to 2020, whereas pharmacy data reflect the situation in 2025. Support for the reorganization of hospital bed functions has been promoted toward the realization of the regional healthcare vision. In 436 hospitals subject to re‑evaluation, the number of acute care beds is projected to decrease from 40,300 in 2017 to 29,100 in 2025, representing an estimated reduction of approximately 28% [28]. Concurrently, financial pressures have precipitated the closure of smaller public hospitals, particularly in low-density rural areas. The number of hospital beds was 1.246 million in 2018, but is estimated to decrease to 1.218 million by 2025 [29]. Consequently, the actual current hospital coverage rates in these regions may be lower than those estimated in this study.” (page 25 lines 447–456)

New References

28.	Ministry of Health, Labour and Welfare. Status of Regional Discussions and Initiatives Related to the Regional Healthcare Vision. 2021. Available from: https://www.mhlw.go.jp/content/10800000/000862586.pdf [cited 2026 Jun 19].

29.	Ministry of Health, Labour and Welfare. About the Regional Healthcare Vision. 2020. Available from: https://www.mhlw.go.jp/content/10800000/000686050.pdf [cited 2026 Jun 19].

**m3 — Policy recommendations need clearer framing
The Discussion now includes two new paragraphs (added in response to Reviewer #1) recommending mobile clinics, transport subsidies, and community-based transportation modes (pp.18-21). These are sensible suggestions well-grounded in the referenced literature. However, they are presented as though they follow directly from the study's empirical findings, when they are in fact literature-based recommendations triggered by the geographic patterns observed. The current framing ("it is necessary to strengthen transportation support…") uses normative language that goes beyond what the data support.
Required action: Minor reframing, add a phrase such as "Drawing on evidence from comparable rural healthcare settings…" or "Based on the broader literature…" at the start of the recommendation paragraph to make clear that these are evidence-informed policy suggestions rather than conclusions directly derivable from this study's spatial data.**
Thank you for this constructive suggestion. We agree that it is important to distinguish between literature-based recommendations and conclusions derived directly from our findings. We have revised the opening sentence as follows.

“Drawing on evidence from comparable rural healthcare settings and the broader literature, complementary measures may help mitigate geographic barriers to healthcare access in rural and mountainous areas with a high concentration of elderly residents. Such measures may include mobile clinics, publicly supported mobile health services, transportation subsidies, and diverse community-based transportation systems. These approaches may complement existing medical institutions and help create an environment in which elderly residents can more readily access necessary medical care within their living areas.” (page 19, line 341– page 20, line 347)

**m4 — Transit proximity does not equal transit connectivity
The public transit accessibility analysis reports the share of population within 400m of a bus stop or train station. This measures 'nearness to a transit node', not 'access to healthcare via transit'. A resident living within 400m of a bus stop is counted as "covered" regardless of whether any route from that stop actually reaches a pharmacy, hospital, or clinic. In the rural regions where the analysis matters most, sparse and infrequent services make this distinction consequential, the 400m proximity metric likely overstates functional transit access to healthcare in these areas.
Required action: Add a sentence to the Limitations section stating explicitly that the transit coverage figures reflect proximity to transit nodes only, and do not capture whether those transit links provide functional connections to healthcare facilities. This framing prevents over-interpretation of transit coverage rates as a proxy for healthcare reachability via public transport.**

Thank you for this important clarification. We agree that proximity to bus stops or train stations does not guarantee effective transit access to healthcare facilities. We have added a statement in the limitation section as follows.

“Additionally, the analysis of public transit accessibility measured only the share of the population living within 400 m of a bus stop or train station, representing nearness to transit nodes rather than actual access to healthcare via transit. This means that residents counted as "covered" may not have functional transit routes connecting them to pharmacies, hospitals, or clinics. In rural regions, where services are sparse and infrequent, this 400 m proximity metric likely overestimates the true accessibility of healthcare through public transportation. Therefore, the transit coverage figures should be interpreted cautiously, as they reflect proximity to transit nodes and do not necessarily correspond to effective healthcare reachability by public transit.” (page 24, line 433–page 25, line 441)
