# Supplementary material for: Accessibility to medical institutions in Aichi Prefecture: A geographic information systems-based analysis
Source: PLoS One. 2026 Aug 26;21(8):e0356748. doi: 10.1371/journal.pone.0356748 (PMC13514043; doi:10.1371/journal.pone.0356748)
Supplement: Peer review history

# Peer review: Decision Letter 0

**DOI:** 10.1371/journal.pone.0356748.r001

**Authors:** Lingye Yao

18 Nov 2025

Dear Dr. Nakamura,

Thank you for submitting your manuscript to PLOS ONE. After careful consideration, we feel that it has merit but does not fully meet PLOS ONE’s publication criteria as it currently stands. Therefore, we invite you to submit a revised version of the manuscript that addresses the points raised during the review process.

Please submit your revised manuscript by Jan 02 2026 11:59PM. If you will need more time than this to complete your revisions, please reply to this message or contact the journal office at plosone@plos.org. When you're ready to submit your revision, log on to https://www.editorialmanager.com/pone/ and select the 'Submissions Needing Revision' folder to locate your manuscript file.

- A rebuttal letter that responds to each point raised by the academic editor and reviewer(s). You should upload this letter as a separate file labeled 'Response to Reviewers'.
- A marked-up copy of your manuscript that highlights changes made to the original version. You should upload this as a separate file labeled 'Revised Manuscript with Track Changes'.
- An unmarked version of your revised paper without tracked changes. You should upload this as a separate file labeled 'Manuscript'.

If you would like to make changes to your financial disclosure, please include your updated statement in your cover letter. Guidelines for resubmitting your figure files are available below the reviewer comments at the end of this letter.

If applicable, we recommend that you deposit your laboratory protocols in protocols.io to enhance the reproducibility of your results. Protocols.io assigns your protocol its own identifier (DOI) so that it can be cited independently in the future. For instructions see: https://journals.plos.org/plosone/s/submission-guidelines#loc-laboratory-protocols. Additionally, PLOS ONE offers an option for publishing peer-reviewed Lab Protocol articles, which describe protocols hosted on protocols.io. Read more information on sharing protocols at https://plos.org/protocols?utm_medium=editorial-email&utm_source=authorletters&utm_campaign=protocols.

We look forward to receiving your revised manuscript.

Kind regards,

Lingye Yao, Ph.D.

Academic Editor

PLOS ONE

Journal Requirements:

When submitting your revision, we need you to address these additional requirements.

1. Please ensure that your manuscript meets PLOS ONE's style requirements, including those for file naming. The PLOS ONE style templates can be found at

https://journals.plos.org/plosone/s/file?id=wjVg/PLOSOne_formatting_sample_main_body.pdf and

https://journals.plos.org/plosone/s/file?id=ba62/PLOSOne_formatting_sample_title_authors_affiliations.pdf

2. We note that the grant information you provided in the ‘Funding Information’ and ‘Financial Disclosure’ sections do not match.

When you resubmit, please ensure that you provide the correct grant numbers for the awards you received for your study in the ‘Funding Information’ section.

3. Thank you for stating in your Funding Statement:

This research was partially supported by JSPS KAKENHI Grant Number, 22K10446.The funders had no role in study design, data collection and analysis, decision to publish, or preparation of the manuscript.

Please provide an amended statement that declares *all* the funding or sources of support (whether external or internal to your organization) received during this study, as detailed online in our guide for authors at http://journals.plos.org/plosone/s/submit-now.  Please also include the statement “There was no additional external funding received for this study.” in your updated Funding Statement.

Please include your amended Funding Statement within your cover letter. We will change the online submission form on your behalf.

4. Please expand the acronym “JSPS” (as indicated in your financial disclosure) so that it states the name of your funders in full.

This information should be included in your cover letter; we will change the online submission form on your behalf.

5. We note that your Data Availability Statement is currently as follows: All relevant data are within the manuscript and its Supporting Information files.

Please confirm at this time whether or not your submission contains all raw data required to replicate the results of your study. Authors must share the “minimal data set” for their submission. PLOS defines the minimal data set to consist of the data required to replicate all study findings reported in the article, as well as related metadata and methods (https://journals.plos.org/plosone/s/data-availability#loc-minimal-data-set-definition).

For example, authors should submit the following data:

- The values behind the means, standard deviations and other measures reported;

- The values used to build graphs;

- The points extracted from images for analysis.

Authors do not need to submit their entire data set if only a portion of the data was used in the reported study.

If your submission does not contain these data, please either upload them as Supporting Information files or deposit them to a stable, public repository and provide us with the relevant URLs, DOIs, or accession numbers. For a list of recommended repositories, please see https://journals.plos.org/plosone/s/recommended-repositories.

If there are ethical or legal restrictions on sharing a de-identified data set, please explain them in detail (e.g., data contain potentially sensitive information, data are owned by a third-party organization, etc.) and who has imposed them (e.g., an ethics committee). Please also provide contact information for a data access committee, ethics committee, or other institutional body to which data requests may be sent. If data are owned by a third party, please indicate how others may request data access.

6. We note that Figures 1 and 3 in your submission contain map images which may be copyrighted. All PLOS content is published under the Creative Commons Attribution License (CC BY 4.0), which means that the manuscript, images, and Supporting Information files will be freely available online, and any third party is permitted to access, download, copy, distribute, and use these materials in any way, even commercially, with proper attribution. For these reasons, we cannot publish previously copyrighted maps or satellite images created using proprietary data, such as Google software (Google Maps, Street View, and Earth). For more information, see our copyright guidelines: http://journals.plos.org/plosone/s/licenses-and-copyright

We require you to either (a) present written permission from the copyright holder to publish these figures specifically under the CC BY 4.0 license, or (b) remove the figures from your submission:

a. You may seek permission from the original copyright holder of Figures 1 and 3 to publish the content specifically under the CC BY 4.0 license.

We recommend that you contact the original copyright holder with the Content Permission Form (http://journals.plos.org/plosone/s/file?id=7c09/content-permission-form.pdf) and the following text:

“I request permission for the open-access journal PLOS ONE to publish XXX under the Creative Commons Attribution License (CCAL) CC BY 4.0 (http://creativecommons.org/licenses/by/4.0/). Please be aware that this license allows unrestricted use and distribution, even commercially, by third parties. Please reply and provide explicit written permission to publish XXX under a CC BY license and complete the attached form.”

Please upload the completed Content Permission Form or other proof of granted permissions as an " "Other" " file with your submission.

In the figure caption of the copyrighted figure, please include the following text: “Reprinted from [ref] under a CC BY license, with permission from [name of publisher], original copyright [original copyright year].”

b. If you are unable to obtain permission from the original copyright holder to publish these figures under the CC BY 4.0 license or if the copyright holder’s requirements are incompatible with the CC BY 4.0 license, please either i) remove the figure or ii) supply a replacement figure that complies with the CC BY 4.0 license. Please check copyright information on all replacement figures and update the figure caption with source information. If applicable, please specify in the figure caption text when a figure is similar but not identical to the original image and is therefore for illustrative purposes only.

The following resources for replacing copyrighted map figures may be helpful:

USGS National Map Viewer (public domain): http://viewer.nationalmap.gov/viewer/

The Gateway to Astronaut Photography of Earth (public domain): http://eol.jsc.nasa.gov/sseop/clickmap/

Maps at the CIA (public domain): https://www.cia.gov/library/publications/the-world-factbook/index.html and https://www.cia.gov/library/publications/cia-maps-publications/index.html

NASA Earth Observatory (public domain): http://earthobservatory.nasa.gov/

Landsat: http://landsat.visibleearth.nasa.gov/

USGS EROS (Earth Resources Observatory and Science (EROS) Center) (public domain): http://eros.usgs.gov/#

Natural Earth (public domain): http://www.naturalearthdata.com/

7. If the reviewer comments include a recommendation to cite specific previously published works, please review and evaluate these publications to determine whether they are relevant and should be cited. There is no requirement to cite these works unless the editor has indicated otherwise.

[Note: HTML markup is below. Please do not edit.]

Reviewers' comments:

Reviewer's Responses to Questions

**Comments to the Author**

1. Is the manuscript technically sound, and do the data support the conclusions?

Reviewer #1: Yes

Reviewer #2: Yes

Reviewer #3: Partly

**********

2. Has the statistical analysis been performed appropriately and rigorously?

Reviewer #1: Yes

Reviewer #2: No

Reviewer #3: N/A

**********

3. Have the authors made all data underlying the findings in their manuscript fully available?

The PLOS Data policy

Reviewer #1: Yes

Reviewer #2: Yes

Reviewer #3: No

**********

4. Is the manuscript presented in an intelligible fashion and written in standard English?

Reviewer #1: Yes

Reviewer #2: Yes

Reviewer #3: Yes

**********

Reviewer #1: 1. The study presents the results of original research. Yes

2. Results reported have not been published elsewhere. No

3. Experiments, statistics, and other analyses are performed to a high technical standard and are described in sufficient detail. No- needs revision as its not clearly identifiable by the reader the 6 indicators and the relationships studied

4. Conclusions are presented in an appropriate fashion and are supported by the data. conclusion section… somewhat… but should be supported by the findings

5. The article is presented in an intelligible fashion and is written in standard English. Yes/ somewhat. But as it is in a descriptive format the indicators and findings are not clear except the two results from the abstract related to urban areas and car ownerships shows easy access compared to rural area and insufficient public transport facilities

6. The research meets all applicable standards for the ethics of experimentation and research integrity. Ethical statement is not clearly identified in the articles

7. The article adheres to appropriate reporting guidelines and community standards for data availability. Yes

This article is timey given the ageing population in Japan and to improve accessibility to healthcare resources or facilities —pharmacies, hospitals, and clinic, to reduce regional disparities as identified across 11 secondary healthcare regions, the official planning units under the Medical Care Act ( ref?).

It is not clear from the abstract or in the paper clearly identified in a table the 6 Accessibility indicators, which the authors compared across regions and age groups.

After figure-1 should be background to the Aichi Prefecture

1. Literature review section is missing. Similar studies could be mentioned from other developing and developed countries with ageing population and healthcare access issues in rural regional areas such as Canada and Australia, similar to Japan.

2. GIS Method used is appropriate, but needs to be clear if any other studies have used such methods in the literature section before moving to the method section. The 6 indicators are still not clearly identifiable. Geographical area for population sampling, 3 types of institution, data source used, is identified.

3. Accessibility indicators ( 6 of them? ) population coverage rate, elderly population coverage rate which are the other 4 indicators? And analytical methods…this could be presented in a table format for clarity ?

4. Study aimed to elucidate regional disparities in accessibility to medical institutions in 67 Aichi Prefecture, Japan, a region characterized by both densely populated urban areas and sparsely 68 populated mountainous areas.

5. And employed Geographic Information Systems (GIS) to quantitatively evaluate the spatial distribution of healthcare resources and accessibility to medical institutions across different regions. The analysis also examined relationships between accessibility and regional characteristics such as population density and the aging rate………….these relationships that the study aims to find...should be mentioned or listed in hypothesis form.

H1- examined relationships between accessibility and regional characteristics such as population density and the aging rate

H2 - between the distribution of healthcare resources and regional characteristic

Any previous studies which included…these two indicators for accessibility – that is population coverage rate and elderly population coverage rate? It could be mentioned .

Authors conducted a comprehensive analysis of accessibility to medical institutions in each 174-region based on indicators such as population coverage rates, population density, aging

rates 175 (percentage of people aged 65 and over), and the distribution of medical resources

Suggestion only: form hypothesis for the 6 indicators ? what are they? Please clarify for the reader, so that the study findings could be generalised and applied to other countries setting with ageing population and regional remote areas, access and transportation issues.

H1- accessibility to medical institutions is positively or negatively related to population coverage rates,

H2-accessibility to medical institutions is positively related to population density,

H3- accessibility to medical institutions is positively related to Pop aging rates

H4- accessibility to medical institutions is positively related to Distribution of medical resources

Please summarise all the findings in a table format as to which indicators ( Accessibility indicators) were compared across regions and age groups were positively or negatively related in terms of accessibility of medical institutions by elderly to Three types of facilities—pharmacies, hospitals, and clinics

The results: These results show that 1- urban areas benefit from concentrated healthcare resources and extensive transit networks, compared to rural and mountainous regions.

2- Although high car ownership may help, but elderly residents remain vulnerable due to mobility restrictions and insufficient public transportation.

Which of the UN- SDGs these findings may help to meet? Please mention in the discussion section.

What are the policy and managerial implication for practice ? in terms of providing easy access to public transportation, and pharmacies, hospitals, and clinics to reduce this regional disparity and access to healthcare facilities and resources, not just for ageing population , but for remote regional areas.

A Major revision is required as mentioned for clarity.

Reviewer #2: Title: Accessibility to medical institutions in Aichi Prefecture: A GIS-based analysis.

Manuscript id: PONE-D-25-46566

reviewer comments

Areal weighting assumes a uniform distribution of population within each source polygon (here: chō-chō-aza units). Large, heterogeneous polygons (e.g., many forest/field areas) will “contribute” population to the buffer simply because of their large area, even though residences are actually far from facilities. As a result, coverage appears higher in rural/mountainous areas with large chō-chō-aza. Conversely, in cities (small polygons, dense buildings), areal weighting tends to be more accurate because the distribution is actually more even on a small scale.

How did the author anticipate edge effects? The author calculated coverage only within the administrative boundaries of secondary healthcare regions (SHRs). Operationally, the facility map was “masked” per SHR, then the population in each SHR was checked to see if it was within a buffer (e.g., 400 m/2 km) of facilities also located in the same SHR. As a result, residents living right on the edge of an SHR, who may be geometrically/distance-wise closer to a facility in a neighboring SHR, will be classified as “uncovered” because the nearest facility is “not counted”.

Data sources have different years (e.g., 2020 census; 2022 bus stops; 2023 railways; 2025 pharmacy list). This can cause time-lag bias in relation to actual current access conditions. When each layer of data comes from a different year, the author is “pasting” together a picture of the city from four different points in time to assess access today. Changes that occur between years, such as population growth, the opening/closing of facilities, bus stop relocations, and the opening of new rail segments, cause the results to be systematically off from actual conditions. How does the author respond to this?

The interpretation that high car ownership “compensates” for limited access is potentially over-optimistic for elderly people with low mobility; the narrative does acknowledge the vulnerability of the elderly, but the authors do not conduct a quantitative analysis of actual modes of transport.

I strongly recommend testing how the results change if the buffer is altered (e.g., 300/500 m; 1/3 km) or if network travel time is used; without this, the robustness of the indicators is not apparent.

The definition of “coverage” = proximity, not “ease of obtaining services.” Without doctor capacity/density, the indicator could be “almost 100% covered” but patients would still wait a long time. The limits of its interpretation need to be emphasized in the abstract and conclusion. (Currently, this is partially acknowledged as a limitation, but it is not emphasized at the beginning).

The claim of “a basis for inferring national trends” is too much of a leap from evidence based on one prefecture (Aichi) to the national level. Inferentially, external validity/transportability is not guaranteed because (i) spatial structure, transport networks, age demographics, and healthcare service arrangements differ between prefectures; (ii) the indicators used (Euclidean distance, fixed buffer, no capacity) may behave differently in metropolitan areas vs. remote rural areas; (iii) there are time lags between data layers that are not uniform.

Reviewer #3: Dear Authors,

This manuscript addresses an important and timely issue, particularly considering aging populations and increasing concern over spatial equity in healthcare access. The manuscript is relatively well structured, the methodology is transparent, and the results effectively demonstrate regional disparities—especially when comparing highly serviced areas such as Nagoya–Owari Central, where 2 km coverage approaches 100%, with regions like Higashimikawa Northern, where access remains significantly limited.

While the manuscript offers valuable empirical insight, several areas require further development before it can be considered for publication.

One key point relates to the theoretical positioning of the study. The introduction references relevant prior work; however, it does not yet fully engage with more recent international literature examining health accessibility inequalities in aging contexts. Over the past five years, this topic has received growing attention, particularly in Western countries where older populations tend to remain in geographically isolated regions as younger populations consolidate in urbanized centers. For example, in the study "Travel-time accessibility and adaptive spatial planning solutions for the healthcare system" (npj Health Systems) analyze similar accessibility gaps and propose adaptive planning strategies in response to demographic aging. Likewise, in the study "Mapping population dynamics at local scales using spatial networks"(Complexity) the authors document emerging dual settlement patterns—urban concentration versus rural aging—which closely parallel the dynamics observed in Aichi Prefecture. Positioning the manuscript more explicitly within this evolving global discussion would significantly enhance its relevance.

Methodologically, although the use of straight-line buffers (400 m and 2 km) is understandable for an initial exploratory assessment, the rationale behind selecting these specific thresholds would benefit from deeper justification. Recent research suggests that accessibility thresholds are most meaningful when connected to travel-time criteria rather than linear distance. For instance, In the previos study the authors propose a 30-minute travel-time threshold as a critical standard, aligned with prior work in spatial accessibility assessment across multiple health systems. Connecting the selected distances to either mobility behavior, national planning benchmarks, or established international standards would strengthen methodological clarity and comparability.

The discussion section could also be further developed. As the authors note, Aichi Prefecture has the highest rate of private vehicle ownership in Japan, which may temporarily compensate for reduced spatial accessibility in rural areas. However, this finding invites deeper interpretation: given the elderly population’s declining driving capacity, it is reasonable to question whether car dependency remains a sustainable long-term assumption. The observed discrepancy in Table 4—where elderly coverage in rural areas is consistently lower—makes this issue particularly relevant.

In addition, the policy implications, though mentioned, could be made more concrete. The results strongly support the relevance of targeted interventions such as telemedicine deployment, integration of mobile health services, expansion of rural transportation schemes, and adaptive spatial planning approaches tailored to aging settlement structures.

Finally, I would like to highlight a technical concern: several figures appear in low resolution and would benefit from being resubmitted with higher visual clarity. Because the maps are central to the paper’s main argument, improving their readability—including clearer legends, labels, and scale indicators—will substantially enhance the manuscript’s accessibility and scientific value.

Thank you again for the opportunity to review this work.

Warm regards,

The revisor

**********

what does this mean?). If published, this will include your full peer review and any attached files.

If you choose “no”, your identity will remain anonymous but your review may still be made public.

**Do you want your identity to be public for this peer review?**  For information about this choice, including consent withdrawal, please see our Privacy Policy

Reviewer #1: No

Reviewer #2: No

Reviewer #3: No

**********

[NOTE: If reviewer comments were submitted as an attachment file, they will be attached to this email and accessible via the submission site. Please log into your account, locate the manuscript record, and check for the action link "View Attachments". If this link does not appear, there are no attachment files.]

To ensure your figures meet our technical requirements, please review our figure guidelines: https://journals.plos.org/plosone/s/figures

You may also use PLOS’s free figure tool, NAAS, to help you prepare publication quality figures: https://journals.plos.org/plosone/s/figures#loc-tools-for-figure-preparation.

NAAS will assess whether your figures meet our technical requirements by comparing each figure against our figure specifications.

# Author comment: Author response to Decision Letter 1

**DOI:** 10.1371/journal.pone.0356748.r002

13 Jan 2026

To reviewer 1

We sincerely thank you for the thorough and constructive review of our manuscript. Your detailed feedback has provided valuable insights that have helped us improve the clarity, rigor, and scholarly contribution of the study. We have addressed each of your comments below, outlining the corresponding revisions and additions made to the manuscript. Our responses are numbered to match your points.

1. The study presents the results of original research. Yes

We have confirmed.

2. Results reported have not been published elsewhere. No

We have confirmed.

3. Experiments, statistics, and other analyses are performed to a high technical standard and are described in sufficient detail. No- needs revision as its not clearly identifiable by the reader the 6 indicators and the relationships studied

Thank you for pointing out this ambiguity. We recognize that our original description was insufficient and may have led to misunderstanding. In our study, the term “accessibility indicator” refers specifically to two measures: population coverage rate and elderly population coverage rate.

The phrase “six indicators” in the manuscript was intended to denote the six population coverage rates calculated for pharmacies, hospitals, and clinics within 400 m and 2 km buffer distances. Because the wording could be misleading, we have revised the relevant sections accordingly.The original text has been updated to read as follows:

“In contrast, the Higashimikawa Northern healthcare region—the least populous region (hereafter referred to as the “rural area”)—showed the lowest coverage for pharmacies and clinics, with differences of more than 15 percentage points compared with other regions. Hospital coverage was also lower; under the 2-km buffer (and similarly under the 3-km buffer), coverage remained more than 20 percentage points below that of other regions (pharmacy 400 m: 20%; pharmacy 2 km: 59%; hospital 400 m: 6%; hospital 2 km: 36%; clinic 400 m: 24%; clinic 2 km: 72%).” (page 14, lines 245–251)

“In contrast, in rural areas, the lowest population coverage rates were observed for hospitals at buffer distances greater than 1 km and for pharmacies and clinics across all buffer distances, revealing clear disparities compared with other regions.” (page 17, lines 300–302)

4. Conclusions are presented in an appropriate fashion and are supported by the data. conclusion section… somewhat… but should be supported by the findings

We appreciate this valuable feedback. We have added the following supporting content.

“The findings revealed a concentration of medical institutions in urban areas and lower coverage rates in rural areas. The Higashimikawa Northern region recorded the lowest values within the 2-km buffer across all facility types: pharmacies (59%), hospitals (36%), and clinics (72%), underscoring pronounced regional disparities in healthcare accessibility. Spatial disparities in proximity to medical institutions were more pronounced among the elderly population, who experienced coverage rates approximately 7% lower than the total population in rural areas, highlighting a persistent accessibility gap for this high-need demographic. Urban areas also demonstrated high public transportation accessibility (96% coverage within 400 m of bus stops and train stations), whereas rural areas fell below 50%, reinforcing reliance on private car access.” (page 23, lines 413–page 24, 421)

5. The article is presented in an intelligible fashion and is written in standard English. Yes/ somewhat. But as it is in a descriptive format the indicators and findings are not clear except the two results from the abstract related to urban areas and car ownerships shows easy access compared to rural area and insufficient public transport facilities

We appreciate your observation regarding the clarity of indicators and findings. We agree that the prior manuscript lacked clarity in presenting indicators and findings. We have restructured the manuscript to ensure indicators and their results are clearly described, as outlined in responses to comments 3 and 4. Please confirm above comments 3 and 4.

6. The research meets all applicable standards for the ethics of experimentation and research integrity. Ethical statement is not clearly identified in the articles

Thank you for pointing out the lack of a clearly identified ethics statement. An Ethics Statement heading has now been added, confirming that no human participants were involved and only publicly available, anonymized, aggregate data were used.

“This study used only publicly available, anonymized, aggregate data from the 2020 Population Census of Japan (via e-Stat [16]) and geospatial datasets from the National Land Numerical Information download service of the Ministry of Land, Infrastructure, Transport and Tourism (MLIT) [15]. No human participants were contacted or surveyed, and no identifiable personal information was collected or analyzed. Accordingly, the study did not constitute “medical and biological research involving human subjects” as defined by Japan’s Ethical Guidelines for Medical and Biological Research Involving Human Subjects [21], and institutional ethical review was not required.” (page 12, lines 210–217)

New references:

15. Ministry of Land, Infrastructure, Transport and Tourism. The National Land Numerical Information download service. Available from: https://nlftp.mlit.go.jp/ksj/ [cited 2025 Dec 23]

16. Statistics Bureau of Japan. e-Stat (Portal Site of Official Statistics of Japan). Available from: https://www.e-stat.go.jp/ [cited 2025 Dec 23]

21. Ministry of Health, Labour and Welfare. Ethical Guidelines for Medical and Biological Research Involving Human Subjects. 2021. Available from: https://www.mhlw.go.jp/content/001457376.pdf [cited 2025 Dec 23]

7. The article adheres to appropriate reporting guidelines and community standards for data availability. Yes

We have confirmed.

8. This article is timey given the ageing population in Japan and to improve accessibility to healthcare resources or facilities —pharmacies, hospitals, and clinic, to reduce regional disparities as identified across 11 secondary healthcare regions, the official planning units under the Medical Care Act ( ref?).

We appreciate your suggestion to cite the relevant legislation for secondary healthcare regions. We have included a citation to the Medical Care Act to define secondary healthcare regions.

(page 7, line 120 and page10, line 180)

New reference:

13. Ministry of Health, Labour and Welfare. Medical Care Act (Act No. 205 of 1948). e-Gov Law Search. 1948. Available from: https://elaws.e-gov.go.jp/document?lawid=323AC0000000205  [cited 2025 Aug 22].

9. It is not clear from the abstract or in the paper clearly identified in a table the 6 Accessibility indicators, which the authors compared across regions and age groups.

Thank you for raising this point again regarding clarity of the six accessibility indicators. This has been addressed as described in our response to comment 3 above. Please confirm above comments 3.

10. After figure-1 should be background to the Aichi Prefecture

We appreciate your recommendation to add background information about Aichi Prefecture. We have included the following:

“Aichi Prefecture covers an area of 5,170 km² and had a population of 7,541,123 in 2020. Its population density was 1,458.6 persons per km², approximately 4.3 times higher than the national average of 338.4 persons per km² [10].” (page 6, line 96–line 98)

11. Literature review section is missing. Similar studies could be mentioned from other developing and developed countries with ageing population and healthcare access issues in rural regional areas such as Canada and Australia, similar to Japan.

Thank you for highlighting the importance of strengthening the literature review. We have added references to related studies from Canada, Australia, and Japan, including research using population coverage rate methodology (Kajimoto et al., 2025).

“In countries such as Canada and Australia, where population aging is advancing similarly to Japan and where rural and remote areas face persistent challenges in healthcare access, Krasniuk et al. [4] and Asante et al. [5] conducted interview-based studies with older adults living in rural regions and highlighted the need to improve access to care services.” (page 4, lines 59–62)

Furthermore, in the Literature Review section, four new references (1, 7, 8, and 9) have been added. (page 3, line 52, page 4, lines 67, 69 and 70)

New references:

1. Balsa-Barreiro J, Morales AJ, Lois-González RC. Mapping Population Dynamics at Local Scales Using Spatial Networks. Complexity. 2021, 8632086. doi: 10.1155/2021/8632086 

4. Krasniuk S, Crizzle AM. Impact of health and transportation on accessing healthcare in older adults living in rural regions. Transp Res Interdiscip Perspect. 2023;21:100882.

5. Asante D, McLachlan CS, Pickles D, Isaac V. Understanding Unmet Care Needs of Rural Older Adults with Chronic Health Conditions: A Qualitative Study. Int J Environ Res Public Health. 2023;20(4):3298. doi:10.3390/ijerph20043298.

7. da Silva CN, Rocha TAH, Amaral PV, Elahi C, Thumé E, Thomaz EBAF, et al. Comprehending the lack of access to maternal and neonatal emergency care: Designing solutions based on a space-time approach. PLoS One. 2020; 15(7): e0235954. doi: 10.1371/journal.pone.0235954.

8. Kajimoto S, Suzuki S, Okada H. Analysis of pharmacy accessibility and function in Wakayama Prefecture using geographic information system. Yakugaku Zasshi. 2025;145(7):639-643. doi:10.1248/yakushi.25-00008.

9. Balsa-Barreiro J, Batista SFA, Hannoun GJ, Menendez M. Travel-time accessibility and adaptive spatial planning solutions for the healthcare system. npj Health Systems. 2025;2:24. doi:10.1038/s44401-025-00028-1.

12. GIS Method used is appropriate, but needs to be clear if any other studies have used such methods in the literature section before moving to the method section.

Thank you for highlighting the importance of strengthening the literature review. We have added references to related studies from Canada, Australia, and Japan, including research using population coverage rate methodology (Kajimoto et al., 2025).

“In Japan, Kajimoto et al. [8] evaluated population coverage rates of pharmacies by secondary healthcare region and examined pharmacy functions in Wakayama Prefecture.” (page 4, lines 68–70)

“To the best of our knowledge, no previous study has comprehensively assessed accessibility to pharmacies, hospitals, and clinics within a region under both walking- and car-based assumptions. In addition, there is a lack of prior research using elderly population coverage rates as an accessibility indicator.” (page 4, line 76– page 5, line 79)

New reference:

8. Kajimoto S, Suzuki S, Okada H. Analysis of pharmacy accessibility and function in Wakayama Prefecture using geographic information system. Yakugaku Zasshi. 2025;145(7):639-643. doi:10.1248/yakushi.25-00008.

13. The 6 indicators are still not clearly identifiable.

Thank you for raising this point again regarding clarity of the six accessibility indicators. This has been addressed as described in our response to comment 3 above. Please confirm above comments 3.

14. Geographical area for population sampling, 3 types of institution, data source used, is identified.

We have confirmed.

15. Accessibility indicators ( 6 of them? ) population coverage rate, elderly population coverage rate which are the other 4 indicators?

Thank you for raising this point again regarding clarity of the six accessibility indicators. This has been addressed as described in our response to comment 3 above. Please confirm above comments 3.

16. And analytical methods…this could be presented in a table format for clarity ?

We appreciate your suggestion to present the analytical methods in a clearer and more understandable format. To improve readability, we have visually summarized the analytical approach in the revised manuscript. Specifically, Figure 2 concisely outlines the analysis workflow and GIS processing steps (buffer creation, areal weighting, coverage calculation), while Table 2 details the data sources used (2020 Census, National Land Numerical Information, etc.).

17. Study aimed to elucidate regional disparities in accessibility to medical institutions in 67 Aichi Prefecture, Japan, a region characterized by both densely populated urban areas and sparsely 68 populated mountainous areas.And employed Geographic Information Systems (GIS) to quantitatively evaluate the spatial distribution of healthcare resources and accessibility to medical institutions across different regions. The analysis also examined relationships between accessibility and regional characteristics such as population density and the aging rate………….these relationships that the study aims to find...should be mentioned or listed in hypothesis form.

H1- examined relationships between accessibility and regional characteristics such as population density and the aging rate

H2 - between the distribution of healthcare resources and regional characteristic

We appreciate your suggestion to clearly state hypotheses related to accessibility and regional characteristics. Based on your input (comment 17,19), we defined and incorporated the following throughout the manuscript:

H1. population coverage rates of medical institutions are positively correlated with population density.

H2. population coverage rates of medical institutions are negatively correlated with the proportion of older adults in the population.

H3. the spatial distribution of healthcare resources is associated with regional characteristics.

The results of H1 and H2 are shown in S3 table.

(page 6, lines 103–107)

(page 17, lines 288–294)

(page 19, line 342–page 20, line 350)

(page 29, lines 516–517)

18. Any previous studies which included…these two indicators for accessibility – that is population coverage rate and elderly population coverage rate? It could be mentioned .

Thank you for highlighting the importance of strengthening the literature review. We have added references to related studies from Canada, Australia, and Japan, including research using population coverage rate methodology (Kajimoto et al., 2025).

“In Japan, Kajimoto et al. [8] evaluated population coverage rates of pharmacies by secondary healthcare region and examined pharmacy functions in Wakayama Prefecture.” (page 4, lines 68–70)

“To the best of our knowledge, no previous study has comprehensively assessed accessibility to pharmacies, hospitals, and clinics within a region under both walking- and car-based assumptions. In addition, there is a lack of prior research using elderly population coverage rates as an accessibility indicator.” (page 4, line 76–page 5, line 79)

19. Authors conducted a comprehensive analysis of accessibility to medical institutions in each 174-region based on indicators such as population coverage rates, population density, aging

rates 175 (percentage of people aged 65 and over), and the distribution of medical resources

Suggestion only: form hypothesis for the 6 indicators ? what are they? Please clarify for the reader, so that the study findings could be generalised and applied to other countries setting with ageing population and regional remote areas, access and transportation issues.

H1- accessibility to medical institutions is positively or negatively related to population coverage rates,

H2-accessibility to medical institutions is positively related to population density,

H3- accessibility to medical institutions is positively related to Pop aging rates

H4- accessibility to medical institutions is positively related to Distribution of medical resources

We appreciate your advice regarding the hypotheses examined in this study. In our analysis, we assessed accessibility to healthcare facilities based on population coverage rates, whereby a higher coverage rate indicates better spatial accessibility. Given this definition, H1 was not tested, as it did not align with our accessibility measure. Similarly, H4 was not tested due to the difficulty in quantitatively evaluating its association with the spatial distribution patterns w

Attachment Submitted filename: 260113_Response to Reviewer3 En.docx

# Peer review: Decision Letter 1

**DOI:** 10.1371/journal.pone.0356748.r003

**Authors:** Lingye Yao

28 Mar 2026

Dear Dr. Nakamura,

Please submit your revised manuscript by May 12 2026 11:59PM. If you will need more time than this to complete your revisions, please reply to this message or contact the journal office at plosone@plos.org. When you're ready to submit your revision, log on to https://www.editorialmanager.com/pone/ and select the 'Submissions Needing Revision' folder to locate your manuscript file.

- A letter that responds to each point raised by the academic editor and reviewer(s). You should upload this letter as a separate file labeled 'Response to Reviewers'.
- A marked-up copy of your manuscript that highlights changes made to the original version. You should upload this as a separate file labeled 'Revised Manuscript with Track Changes'.
- An unmarked version of your revised paper without tracked changes. You should upload this as a separate file labeled 'Manuscript'.

If you would like to make changes to your financial disclosure, please include your updated statement in your cover letter. Guidelines for resubmitting your figure files are available below the reviewer comments at the end of this letter.

If applicable, we recommend that you deposit your laboratory protocols in protocols.io to enhance the reproducibility of your results. Protocols.io assigns your protocol its own identifier (DOI) so that it can be cited independently in the future. For instructions see: https://journals.plos.org/plosone/s/submission-guidelines#loc-laboratory-protocols. Additionally, PLOS ONE offers an option for publishing peer-reviewed Lab Protocol articles, which describe protocols hosted on protocols.io. Read more information on sharing protocols at https://plos.org/protocols?utm_medium=editorial-email&utm_source=authorletters&utm_campaign=protocols.

As the corresponding author, your ORCID iD is verified in the submission system and will appear in the published article. PLOS supports the use of ORCID, and we encourage all coauthors to register for an ORCID iD and use it as well. Please encourage your coauthors to verify their ORCID iD within the submission system before final acceptance, as unverified ORCID iDs will not appear in the published article. *Only*  the individual author can complete the verification step; PLOS staff *cannot*  verify ORCID iDs on behalf of authors.

We look forward to receiving your revised manuscript.

Kind regards,

Lingye Yao, Ph.D.

Academic Editor

PLOS One

Journal Requirements:

If the reviewer comments include a recommendation to cite specific previously published works, please review and evaluate these publications to determine whether they are relevant and should be cited. There is no requirement to cite these works unless the editor has indicated otherwise.

Please review your reference list to ensure that it is complete and correct. If you have cited papers that have been retracted, please include the rationale for doing so in the manuscript text, or remove these references and replace them with relevant current references. Any changes to the reference list should be mentioned in the rebuttal letter that accompanies your revised manuscript. If you need to cite a retracted article, indicate the article’s retracted status in the References list and also include a citation and full reference for the retraction notice.

[Note: HTML markup is below. Please do not edit.]

Reviewers' comments:

Reviewer's Responses to Questions

**Comments to the Author**

Reviewer #1: All comments have been addressed

Reviewer #3: All comments have been addressed

**********

2. Is the manuscript technically sound, and do the data support the conclusions?

Reviewer #1: Yes

Reviewer #3: Yes

**********

3. Has the statistical analysis been performed appropriately and rigorously?

Reviewer #1: Yes

Reviewer #3: Yes

**********

4. Have the authors made all data underlying the findings in their manuscript fully available?

The PLOS Data policy

Reviewer #1: No

Reviewer #3: Yes

**********

5. Is the manuscript presented in an intelligible fashion and written in standard English?

Reviewer #1: Yes

Reviewer #3: Yes

**********

Reviewer #1: Given the aging population in Japan and many developed countries there is inequality in access to the three healthcare facilities in regional, rural and remote areas.

This study investigated the geographic accessibility of medical institutions in Aichi Prefecture, Japan, using Geographic Information Systems (GIS), in three facilities—pharmacies, hospitals, and clinics—with a focus on Accessibility & Catchment areas

The study results showed that urban areas benefit from concentrated healthcare resources and extensive transit networks, compared to rural and mountainous regions which remained

underserved.

Authors need to show in a framework as to how geographic and demographic perspectives can be integrated into healthcare policy to support community

care and sustainable resource allocation for senior citizens

Discussion needs to be elaborated, and authors need to come up with recommendation and a framework to show how rural-urban disparities in access to

three healthcare institutions—pharmacies, hospitals, and clinics—can be improved via better policy and resource allocation for the elderly.

What is the future research direction in terms of asking/interviewing the elderly through interviews and personally administered survey with the elderly population living in this 11 geographical areas, as to what they need to make life /old aged people conformable in context of medical facilities access, availability, and affordability as well as other elderly needs for real policy impact.

Reviewer #3: After reviewing the revised version of the manuscript and the authors’ detailed responses to the previous round of comments, I consider that the main concerns raised during the initial review have been satisfactorily addressed. The authors have made substantive revisions that improve the clarity, methodological transparency, and overall robustness of the study.

In my view, the manuscript has significantly improved and now presents a clearer and more solid contribution. I therefore recommend that the manuscript be considered suitable for publication.

**********

what does this mean?). If published, this will include your full peer review and any attached files.

If you choose “no”, your identity will remain anonymous but your review may still be made public.

**Do you want your identity to be public for this peer review?**  For information about this choice, including consent withdrawal, please see our Privacy Policy

Reviewer #1: No

Reviewer #3: No

**********

[NOTE: If reviewer comments were submitted as an attachment file, they will be attached to this email and accessible via the submission site. Please log into your account, locate the manuscript record, and check for the action link "View Attachments". If this link does not appear, there are no attachment files.]

To ensure your figures meet our technical requirements, please review our figure guidelines: https://journals.plos.org/plosone/s/figures

You may also use PLOS’s free figure tool, NAAS, to help you prepare publication quality figures: https://journals.plos.org/plosone/s/figures#loc-tools-for-figure-preparation.

NAAS will assess whether your figures meet our technical requirements by comparing each figure against our figure specifications.

# Author comment: Author response to Decision Letter 2

**DOI:** 10.1371/journal.pone.0356748.r004

9 Apr 2026

Response to Reviewer #1

We wish to express our sincere gratitude for your constructive and insightful comments, which have greatly contributed to improving the clarity, depth, and policy relevance of our manuscript. Each of your suggestions has been carefully considered, and corresponding revisions have been incorporated to strengthen the discussion, provide practical frameworks for integrating geographic and demographic perspectives into healthcare policy, address rural–urban disparities in access to pharmacies, hospitals, and clinics for elderly populations, and highlight directions for future research involving direct engagement with elderly residents. The following presents our detailed, point-by-point responses, with page and line numbers indicating the exact locations of the modifications in the revised version.

1. Authors need to show in a framework as to how geographic and demographic perspectives can be integrated into healthcare policy to support community care and sustainable resource allocation for senior citizens.

We sincerely thank you for this valuable comment. We agree that systematically integrating geographic and demographic perspectives into healthcare policy is essential to strengthen community care and ensure sustainable allocation of medical resources for senior citizens. Based on your suggestion, we have added the following explanation to the Discussion section:

“Geographic and demographic perspectives can be systematically integrated into healthcare policies to support community care and sustainable allocation of medical resources for elderly individuals. This integration should focus on improving access in underserved rural and mountainous areas and promoting multimodal transportation systems along with healthcare delivery methods suited to local needs. In addition, regional healthcare planning should use specific spatial analysis measures, such as population and elderly population coverage rates, to design targeted strategies that address the unique demographic features and mobility limitations of each area.” (page 21, lines 374–380)

2. Discussion needs to be elaborated, and authors need to come up with recommendation and a framework to show how rural-urban disparities in access to three healthcare institutions—pharmacies, hospitals, and clinics—can be improved via better policy and resource allocation for the elderly.

We sincerely appreciate your insightful suggestion to strengthen the Discussion section by focusing on policy recommendations and a framework to reduce rural–urban disparities in access to pharmacies, hospitals, and clinics for elderly populations. We have added the following explanation to the Discussion section:

“To address these disparities, strategic reallocation and functional enhancement of medical facilities while considering the spatial distribution of the elderly population in each region are required. Specifically, in rural and mountainous areas with a high concentration of elderly residents, the introduction of mobile clinics and publicly supported mobile health services to complement existing medical institutions is expected to be effective. Furthermore, to alleviate mobility constraints among the elderly, it is necessary to strengthen transportation support by enhancing public transportation services and expanding transportation cost subsidy programs, while promoting the development of diverse, community-based transportation modes. These measures can create an environment where elderly individuals can access the necessary medical care within their living areas more readily, thereby reducing geographic disparities in healthcare accessibility between urban and rural regions. In healthcare policy planning, positioning such comprehensive interventions and implementing flexible resource allocation tailored to regional characteristics are of critical importance.” (page 18, line 320–page 19, line 332)

3. What is the future research direction in terms of asking/interviewing the elderly through interviews and personally administered survey with the elderly population living in this 11 geographical areas, as to what they need to make life /old aged people conformable in context of medical facilities access, availability, and affordability as well as other elderly needs for real policy impact.

We appreciate your insightful suggestion to incorporate direct input from elderly residents in future research. We have added the following statement to the Discussion section:

“Future studies should also incorporate interviews and surveys with residents, including elderly individuals, in the 11 secondary healthcare regions to capture diverse needs and preferences in relation to healthcare access and related challenges. Integrating the perspectives of all community members will provide comprehensive insights into obstacle experienced in daily life and support the development of effective, user-centered policies aimed at improving healthcare accessibility and overall well-being across various geographical settings.” (page 24, lines 424–429)

-----

Response to Reviewer #3

We sincerely thank you for your thorough re-evaluation of our manuscript and your positive assessment. We greatly appreciate your recognition of the substantive revisions made to improve the clarity, methodological transparency, and robustness of our study. Your constructive comments have been invaluable throughout the review process, and we are grateful that you consider the manuscript now suitable for publication.

Attachment Submitted filename: Response to reviewer 1.docx

# Peer review: Decision Letter 2

**DOI:** 10.1371/journal.pone.0356748.r005

**Authors:** Lingye Yao

3 Jun 2026

Dear Dr. Nakamura,

Please submit your revised manuscript by Jul 18 2026 11:59PM. If you will need more time than this to complete your revisions, please reply to this message or contact the journal office at plosone@plos.org. When you're ready to submit your revision, log on to https://www.editorialmanager.com/pone/ and select the 'Submissions Needing Revision' folder to locate your manuscript file.

- A letter that responds to each point raised by the academic editor and reviewer(s). You should upload this letter as a separate file labeled 'Response to Reviewers'.
- A marked-up copy of your manuscript that highlights changes made to the original version. You should upload this as a separate file labeled 'Revised Manuscript with Track Changes'.
- An unmarked version of your revised paper without tracked changes. You should upload this as a separate file labeled 'Manuscript'.

If you would like to make changes to your financial disclosure, please include your updated statement in your cover letter. Guidelines for resubmitting your figure files are available below the reviewer comments at the end of this letter.

If applicable, we recommend that you deposit your laboratory protocols in protocols.io to enhance the reproducibility of your results. Protocols.io assigns your protocol its own identifier (DOI) so that it can be cited independently in the future. For instructions see: https://journals.plos.org/plosone/s/submission-guidelines#loc-laboratory-protocols. Additionally, PLOS ONE offers an option for publishing peer-reviewed Lab Protocol articles, which describe protocols hosted on protocols.io. Read more information on sharing protocols at https://plos.org/protocols?utm_medium=editorial-email&utm_source=authorletters&utm_campaign=protocols.

As the corresponding author, your ORCID iD is verified in the submission system and will appear in the published article. PLOS supports the use of ORCID, and we encourage all coauthors to register for an ORCID iD and use it as well. Please encourage your coauthors to verify their ORCID iD within the submission system before final acceptance, as unverified ORCID iDs will not appear in the published article. *Only*  the individual author can complete the verification step; PLOS staff *cannot*  verify ORCID iDs on behalf of authors.

We look forward to receiving your revised manuscript.

Kind regards,

Lingye Yao, Ph.D.

Academic Editor

PLOS One

**Journal Requirements:**

1. If the reviewer comments include a recommendation to cite specific previously published works, please review and evaluate these publications to determine whether they are relevant and should be cited. There is no requirement to cite these works unless the editor has indicated otherwise.

2. Please review your reference list to ensure that it is complete and correct. If you have cited papers that have been retracted, please include the rationale for doing so in the manuscript text, or remove these references and replace them with relevant current references. Any changes to the reference list should be mentioned in the rebuttal letter that accompanies your revised manuscript. If you need to cite a retracted article, indicate the article’s retracted status in the References list and also include a citation and full reference for the retraction notice.

[Note: HTML markup is below. Please do not edit.]

Reviewers' comments:

Reviewer's Responses to Questions

**Comments to the Author**

Reviewer #4: All comments have been addressed

Reviewer #5: All comments have been addressed

**********

2. Is the manuscript technically sound, and do the data support the conclusions?

Reviewer #4: Yes

Reviewer #5: Partly

**********

3. Has the statistical analysis been performed appropriately and rigorously?

Reviewer #4: Yes

Reviewer #5: N/A

**********

4. Have the authors made all data underlying the findings in their manuscript fully available?

The PLOS Data policy

Reviewer #4: Yes

Reviewer #5: Yes

**********

5. Is the manuscript presented in an intelligible fashion and written in standard English?

Reviewer #4: Yes

Reviewer #5: Yes

**********

Reviewer #4: In this revision, the authors have addressed all concerns and the manuscript can be accepted for publication.

Reviewer #5: The paper uses GIS buffer analysis (400m walk / 2km drive) with areal weighting on 2020 Census chō-chō-aza units to measure population and elderly population coverage rates for three facility types (pharmacies, hospitals, clinics) across 11 secondary healthcare regions in Aichi Prefecture, Japan. Also measures public transit accessibility via 400m buffers around bus stops and train stations. Sensitivity analyses test buffer radii from 300m to 3km. Simple regression tests correlations between coverage rates and (a) population density and (b) aging rate. Software: ArcGIS Pro 3.4.0.

Criterion 1 — Original research

The simultaneous analysis of three facility types under both walking and driving assumptions, using elderly population coverage rate as an indicator, has not been done for Aichi Prefecture (or apparently the broader region). The authors' novelty claim is reasonable and not overstated.

Criterion 2 — Not published elsewhere

Appears to be original work from the Drug Informatics lab at Gifu Pharmaceutical University.

Criterion 3 — Technical standard and methodological detail

Partially met — several issues require attention (see Major and Minor Concerns below).

Core methodology (Euclidean buffer analysis + areal weighting + census data) is appropriate and well-described. Data sources are fully cited and publicly accessible. Sensitivity analysis across six buffer distances is a genuine strength. However:

- Regression analyses use only N=11 data points (one per secondary healthcare region), giving very low statistical power and high leverage for individual outliers.

- Euclidean buffers are cited against the Walk Score 5-min walk standard, but Walk Score uses pedestrian network distances; this is methodologically inconsistent.

- Geocoding success rate for pharmacy addresses is unreported.

- Hospital/clinic location data is from 2020 (MLIT) while pharmacy data is from 2025, creating a 5-year temporal asymmetry within the same analysis.

Criterion 4 — Conclusions supported by data

Mostly met. The core descriptive findings (urban-rural disparity, elderly coverage gap, transit coverage gap) are directly supported by the data and clearly presented. The regression findings are appropriately cautious regarding the aging rate correlation. One concern: the policy recommendations (mobile clinics, transport subsidies, etc.) are imported from the literature rather than derived from this study's empirical findings; this should be more explicitly framed as narrative synthesis rather than evidence-based conclusions from the present data.

Criterion 5 — Standard English

Mostly met. Writing is generally clear and accessible to non-specialists. No notable language issues.

Criterion 6 — Ethics

N/A

Criterion 7 — Reporting guidelines and data availability

Partially met — minor gaps.

Data availability statement ("All relevant data are within the manuscript and its Supporting Information files") is acceptable given that all source data come from public repositories (e-Stat, MLIT, Tokai-Hokuriku Bureau). However, the geocoded pharmacy point layer (the processed output from the CSV Address Matching Service) is a derived dataset that does not exist in any public repository. Ideally this derived dataset should be deposited. Supporting Tables S1-S3 cover the coverage rates and correlations.

No formal reporting checklist (e.g., STROBE for observational studies) is provided. While STROBE is primarily designed for epidemiological studies, an ecological GIS study using population data falls within its scope; at least, the authors should check and confirm whether any relevant checklist applies to this study type.

Major concerns

M1 — Regression analyses with N=11: insufficient power and outlier sensitivity

The simple regression analyses comparing population coverage rates against population density and aging rate are conducted with N=11 observations (one per secondary healthcare region). At this sample size, the analyses have extremely low statistical power and R² values are easily driven by one or two outliers. The contrast between Higashimikawa Northern (density 50/km², aging rate 38.5%) and Nagoya-Owari Central (density 6,785/km², aging rate 24.2%) is so extreme that these two regions likely account for most of the observed variance.

Required action: The authors should either (a) explicitly acknowledge in the text that the regression results are highly sensitive to outlier regions and should be treated as descriptive rather than inferential, or (b) report the change in R² when the two extreme regions (Higashimikawa Northern and Nagoya-Owari Central) are excluded, to show whether the relationships hold in the mid-range regions. The current text presents R²>0.70 as a "relatively strong positive correlation" without this caveat; this is overconfident given the sample size.

M2 — Euclidean buffer vs. Walk Score network distance: methodological inconsistency

The 400m walking buffer is justified by citing the Walk Score methodology (ref 18: this seems a methodology webpage, not a peer-reviewed source), which assigns full walkability credit up to 400 m (0.25 miles, ~5-min walk) before applying a distance-decay function. Walk Score, however, calculates distances using the actual pedestrian street network, not straight-line (Euclidean) distance. The authors acknowledge Euclidean distance as a general spatial limitation elsewhere in the paper (citing Boscoe et al. 2012 for driving accessibility), but they do not address the specific inconsistency that the citation they invoke to justify the 400m threshold, Walk Score, itself relies on network distances. The two approaches are not interchangeable: the detour index (ratio of network to Euclidean distance) consistently exceeds 1.0 in urban street grids and can be substantially higher in mountainous terrain, meaning the Euclidean 400m buffer overstates walkable coverage relative to what the Walk Score standard actually implies, with the effect most pronounced in the rural areas where access gaps are largest.

Required action: The authors should revise the Walk Score citation language to make clear that their Euclidean buffer approximates but does not replicate the Walk Score methodology, and add a sentence acknowledging this specific inconsistency and its directional effect on coverage estimates in mountainous versus urban settings.

Minor concerns

m1 — Geocoding success rate not reported

Pharmacy locations were derived by geocoding 3,962 addresses (of which 3,628 are in Aichi Prefecture) using the University of Tokyo CSV Address Matching Service. The geocoding success rate (proportion of addresses successfully matched) and the spatial precision of matches are not reported. Failed matches or low-confidence matches would create gaps in the pharmacy coverage map that are invisible in the current analysis.

Required action: Report the geocoding success rate (number/percentage of addresses successfully geocoded) and the precision level of matches (e.g., address-level vs. street-level vs. area-level). If any addresses failed geocoding, state what proportion this represents.

m2 — Temporal mismatch between data layers is asymmetric

The authors acknowledge the time-lag limitation (p.23, lines 406-413), the study combines 2020 Census population data with 2022 bus stop data, 2023 railway data, and a 2025 pharmacy list. This is reasonable given data availability constraints. However, the discussion does not note that this mismatch is directionally asymmetric: pharmacy data is from 2025 (most current), while hospital/clinic data is from 2020 (MLIT). Between 2020 and 2025, Japan's Regional Healthcare Vision has driven national bed restructuring (targeting a 30% reduction in acute-phase hospital beds), and mounting financial pressures on smaller public hospitals have contributed to closures nationally. The hospital layer (321 hospitals in Aichi Prefecture, 2020) may therefore overstate current availability, particularly in lower-density areas where smaller hospitals are more financially vulnerable.

Required action: Add a sentence to the limitations paragraph explicitly noting that the hospital/clinic data (2020) is 5 years older than the pharmacy data (2025), and that hospital restructuring between these years may mean that current hospital coverage rates in rural areas are lower than reported.

m3 — Policy recommendations need clearer framing

The Discussion now includes two new paragraphs (added in response to Reviewer #1) recommending mobile clinics, transport subsidies, and community-based transportation modes (pp.18-21). These are sensible suggestions well-grounded in the referenced literature. However, they are presented as though they follow directly from the study's empirical findings, when they are in fact literature-based recommendations triggered by the geographic patterns observed. The current framing ("it is necessary to strengthen transportation support…") uses normative language that goes beyond what the data support.

Required action: Minor reframing, add a phrase such as "Drawing on evidence from comparable rural healthcare settings…" or "Based on the broader literature…" at the start of the recommendation paragraph to make clear that these are evidence-informed policy suggestions rather than conclusions directly derivable from this study's spatial data.

m4 — Transit proximity does not equal transit connectivity

The public transit accessibility analysis reports the share of population within 400m of a bus stop or train station. This measures 'nearness to a transit node', not 'access to healthcare via transit'. A resident living within 400m of a bus stop is counted as "covered" regardless of whether any route from that stop actually reaches a pharmacy, hospital, or clinic. In the rural regions where the analysis matters most, sparse and infrequent services make this distinction consequential, the 400m proximity metric likely overstates functional transit access to healthcare in these areas.

Required action: Add a sentence to the Limitations section stating explicitly that the transit coverage figures reflect proximity to transit nodes only, and do not capture whether those transit links provide functional connections to healthcare facilities. This framing prevents over-interpretation of transit coverage rates as a proxy for healthcare reachability via public transport.

**********

what does this mean?). If published, this will include your full peer review and any attached files.

If you choose “no”, your identity will remain anonymous but your review may still be made public.

**Do you want your identity to be public for this peer review?**  For information about this choice, including consent withdrawal, please see our Privacy Policy

Reviewer #4: **Yes:** Kiki Adhinugraha

Reviewer #5: No

**********

[NOTE: If reviewer comments were submitted as an attachment file, they will be attached to this email and accessible via the submission site. Please log into your account, locate the manuscript record, and check for the action link "View Attachments". If this link does not appear, there are no attachment files.]

To ensure your figures meet our technical requirements, please review our figure guidelines: https://journals.plos.org/plosone/s/figures

You may also use PLOS’s free figure tool, NAAS, to help you prepare publication quality figures: https://journals.plos.org/plosone/s/figures#loc-tools-for-figure-preparation.

NAAS will assess whether your figures meet our technical requirements by comparing each figure against our figure specifications.

# Author comment: Author response to Decision Letter 3

**DOI:** 10.1371/journal.pone.0356748.r006

24 Jun 2026

Response to Reviewer #4

We sincerely thank you for the positive evaluation and kind comments.

We appreciate your acknowledgement that our revisions have addressed all previous concerns, and we are grateful for your support in recommending the manuscript for publication.

-----

Response to Reviewer #5

We sincerely thank you for the thorough and constructive comments on our manuscript, including both the positive assessments of novelty and methodological strengths, and the detailed suggestions for improvement.

We have carefully addressed each major and minor concern in this revision, as detailed below.

Criterion 1–6

We have confirmed.

Criterion 7 — Reporting guidelines and data availability

Partially met — minor gaps.

Data availability statement ("All relevant data are within the manuscript and its Supporting Information files") is acceptable given that all source data come from public repositories (e-Stat, MLIT, Tokai-Hokuriku Bureau). However, the geocoded pharmacy point layer (the processed output from the CSV Address Matching Service) is a derived dataset that does not exist in any public repository. Ideally this derived dataset should be deposited. Supporting Tables S1-S3 cover the coverage rates and correlations.

Regarding the request to deposit the derived dataset (i.e., the geocoded pharmacy point layer produced from the CSV Address Matching Service), we acknowledge that this dataset does not currently exist in any public repository. While we are not making this dataset directly available as a Supplementary Table at this stage, we are willing to provide it upon reasonable request to the corresponding author, in accordance with data-sharing policies. This approach ensures that all source data remain accessible while respecting any potential usage constraints of the derived geocoded layer. We added the Data availability section as follows.

“Data availability

All relevant source data used in this study are publicly available from official repositories: Ministry of Land, Infrastructure, Transport and Tourism (MLIT) datasets [15], e-Stat [16], and the Tokai-Hokuriku Regional Bureau[17]. The minimal data set is fully disclosed in Table 2. Supporting Tables S1-S4, included as supplementary information, provide detailed coverage rates and correlations related to the datasets.

The geocoded pharmacy point layer, which is a derived dataset produced using the CSV Address Matching Service [14], does not currently exist in any public repository. While this derived dataset is not included as a supplementary file, it is available from the corresponding author, M.N., upon reasonable request, subject to data-sharing policies and any usage restrictions.” (page 28, lines 500–509)

No formal reporting checklist (e.g., STROBE for observational studies) is provided. While STROBE is primarily designed for epidemiological studies, an ecological GIS study using population data falls within its scope; at least, the authors should check and confirm whether any relevant checklist applies to this study type.

With respect to the reporting checklist, we reviewed available guidelines. We did not identify any checklist that fully matches the scope and methodological characteristics of this ecological GIS study. Although some items in existing checklists are not applicable, we elected to use the STROBE Statement—Checklist of items that should be included in reports of cross-sectional studies as the most relevant framework. This completed checklist has been submitted as an "others" file entitled “for the reviewer #5 STROBE” for editorial assessment.

Major concerns

M1 — Regression analyses with N=11: insufficient power and outlier sensitivity

The simple regression analyses comparing population coverage rates against population density and aging rate are conducted with N=11 observations (one per secondary healthcare region). At this sample size, the analyses have extremely low statistical power and R² values are easily driven by one or two outliers. The contrast between Higashimikawa Northern (density 50/km², aging rate 38.5%) and Nagoya-Owari Central (density 6,785/km², aging rate 24.2%) is so extreme that these two regions likely account for most of the observed variance.

Required action: The authors should either (a) explicitly acknowledge in the text that the regression results are highly sensitive to outlier regions and should be treated as descriptive rather than inferential, or (b) report the change in R² when the two extreme regions (Higashimikawa Northern and Nagoya-Owari Central) are excluded, to show whether the relationships hold in the mid-range regions. The current text presents R²>0.70 as a "relatively strong positive correlation" without this caveat; this is overconfident given the sample size.

Thank you for this insightful comment. We fully agree that the regression analysis in our study, with a small sample size (N = 11), is highly sensitive to extreme values and therefore requires cautious interpretation. Accordingly, we have substantially revised the wording in the relevant sections of the manuscript to clearly state that these regressions should be regarded as descriptive explorations rather than inferential analyses. In addition, we conducted a sensitivity analysis excluding the two extreme regions (Higashimikawa Northern and Nagoya-Owari Central), and reported the results in Table S4. The manuscript has been revised as follows.

“S3 Table presents the correlations between population coverage rates and population density, as well as between population coverage rates and the aging rate. In the full dataset of 11 secondary healthcare regions, positive correlations with R² values greater than 0.70 were observed between population coverage rates and population density for pharmacies and clinics at 300 m and 400 m, and for hospitals at 300 m, 400 m, 500 m, and 1 km. Negative correlations with R² values greater than 0.70 were also observed between population coverage rates and the aging rate for pharmacies and clinics at 2 km and 3 km. However, because these regression analyses were based on only 11 observations, the results may be sensitive to extreme regions. Therefore, we conducted a sensitivity analysis excluding Nagoya–Owari Central and Higashimikawa Northern, which represented the highest-density urban region and the lowest-density rural region, respectively (S4 Table). After excluding these two regions, the previously observed correlations were attenuated, and the correlations between population coverage rates and the aging rate fell below an R² of 0.70 across all facility types and buffer distances. These findings indicate that the regression analyses should be interpreted as descriptive explorations of regional trends rather than inferential evidence of consistent associations.” (page 17, line 299–page 18, line 313)

“In S3 Table, positive correlations (R² > 0.70) between population coverage rates and population density and negative correlations (R² > 0.70) between population coverage rates and the aging rate were observed in the full dataset. However, due to the small sample size (n = 11), much of the observed variation may have been influenced by two extreme regions. In S4 Table, which excludes Nagoya–Owari Central and Higashimikawa Northern, the correlations observed in the full dataset were attenuated. Therefore, the relationships between population coverage rates and population density, as well as between population coverage rates and the aging rate, should be interpreted with caution. In this study, these regression analyses are better viewed as descriptive explorations of regional trends rather than inferential tests providing strong evidence of causal or consistent relationships across regions. Overall, while a tendency toward higher population coverage rates with increasing population density was observed, there was no evidence supporting a simple and consistent association between the aging rate and population coverage rates across the secondary healthcare regions.” (page 21, lines 367–379)

“S4 Table. Correlations between population coverage rates and population density and between population coverage rates and aging rates (excluding regions: Higashimikawa Northern (lowest population, rural) and Nagoya-Owari Central (highest population, urban)).” (page 33, lines 603–605)

M2 — Euclidean buffer vs. Walk Score network distance: methodological inconsistency

The 400m walking buffer is justified by citing the Walk Score methodology (ref 18: this seems a methodology webpage, not a peer-reviewed source), which assigns full walkability credit up to 400 m (0.25 miles, ~5-min walk) before applying a distance-decay function. Walk Score, however, calculates distances using the actual pedestrian street network, not straight-line (Euclidean) distance. The authors acknowledge Euclidean distance as a general spatial limitation elsewhere in the paper (citing Boscoe et al. 2012 for driving accessibility), but they do not address the specific inconsistency that the citation they invoke to justify the 400m threshold, Walk Score, itself relies on network distances. The two approaches are not interchangeable: the detour index (ratio of network to Euclidean distance) consistently exceeds 1.0 in urban street grids and can be substantially higher in mountainous terrain, meaning the Euclidean 400m buffer overstates walkable coverage relative to what the Walk Score standard actually implies, with the effect most pronounced in the rural areas where access gaps are largest.

Required action: The authors should revise the Walk Score citation language to make clear that their Euclidean buffer approximates but does not replicate the Walk Score methodology, and add a sentence acknowledging this specific inconsistency and its directional effect on coverage estimates in mountainous versus urban settings.

Thank you for pointing out this methodological inconsistency. We agree that Euclidean distance and Walk Score’s network distance calculation are not interchangeable. To address this, we have revised the text to clarify that our 400m Euclidean buffer approximates but does not replicate the Walk Score methodology. We also added a statement noting that Euclidean buffers likely overestimate walkable coverage, particularly in rural and mountainous areas, due to higher detour index values. We modified the method section and added an explanation to the limitation section as follows.

“The 400-m buffer was used as an approximation of, but did not replicate, the Walk Score methodology, which defines a 5-min walk (approximately 0.25 miles) as the upper threshold for walkable access [18].” (page 10, lines 176–178)

“Furthermore, the 400-m walking buffer adopted in this study approximates, but does not replicate, the Walk Score methodology [18], which evaluates a distance of 400 m (approximately 0.25 miles, or a 5-minute walk) as a complete walkable distance and applies a distance-decay function. However, whereas Walk Score calculates distances based on the actual pedestrian street network, this study used Euclidean (straight-line) distance. These two distance measurement methods are not interchangeable, and the ratio of network distance to Euclidean distance (circuity factor) is generally greater than 1.0; this ratio may be even larger in mountainous terrain. Therefore, the 400-m Euclidean distance buffer applied in this study likely overestimates the actual walkable range implied by the Walk Score standard, particularly in rural areas where access disparities are substantial.” (page 23, line 415– page 24, line 424)

Minor concerns

m1 — Geocoding success rate not reported

Pharmacy locations were derived by geocoding 3,962 addresses (of which 3,628 are in Aichi Prefecture) using the University of Tokyo CSV Address Matching Service. The geocoding success rate (proportion of addresses successfully matched) and the spatial precision of matches are not reported. Failed matches or low-confidence matches would create gaps in the pharmacy coverage map that are invisible in the current analysis.

Required action: Report the geocoding success rate (number/percentage of addresses successfully geocoded) and the precision level of matches (e.g., address-level vs. street-level vs. area-level). If any addresses failed geocoding, state what proportion this represents.

Thank you for this valuable comment. We agree that geocoding success rate and precision levels are important for interpreting the results. We have now included these details in the methods section:

“All 3,962 pharmacy addresses were successfully geocoded using the CSV Address Matching Service. These addresses were geocoded with varying levels of spatial precision. Among the 3,628 pharmacies located within Aichi Prefecture, 3,250 (approximately 89.7%) were matched to the most detailed geographic units corresponding to city blocks and individual lot numbers. Additionally, 294 addresses were matched to subdivisions roughly equivalent to neighborhood sections (chōme and kōaza), 79 to larger administrative subdivisions similar to wards or local districts (ōaza), 1 to the ward level within designated cities, and 4 to the broader municipality level (city, town, or village). For the entire dataset of 3,962 pharmacies, 3,525 (about 89.0%) were geocoded at the finest level of city block and lot number, 313 at neighborhood subdivision level, 119 at larger district level, 1 at ward level, and 4 at municipality level.” (page 8, line 139–page 9, line 149)

m2 — Temporal mismatch between data layers is asymmetric

The authors acknowledge the time-lag limitation (p.23, lines 406-413), the study combines 2020 Census population data with 2022 bus stop data, 2023 railway data, and a 2025 pharmacy list. This is reasonable given data availability constraints. However, the discussion does not note that this mismatch is directionally asymmetric: pharmacy data is from 2025 (most current), while hospital/clinic data is from 2020 (MLIT). Between 2020 and 2025, Japan's Regional Healthcare Vision has driven national bed restructuring (targeting a 30% reduction in acute-phase hospital beds), and mounting financial pressures on smaller public hospitals have contributed to closures nationally. The hospital layer (321 hospitals in Aichi Prefecture, 2020) may therefore overstate current availability, particularly in lower-density areas where smaller hospitals are more financially vulnerable.

Required action: Add a sentence to the limitations paragraph explicitly noting that the hospital/clinic data (2020) is 5 years older than the pharmacy data (2025), and that hospital restructuring between these years may mean that current hospital coverage rates in rural areas are lower than reported.

Thank you for this important observation. We agree that hospital and clinic data (2020) are five years older than pharmacy data (2025), and that restructuring between these years may reduce current hospital availability, especially in rural areas. We have added this statement to the limitations section.

“Moreover, this temporal mismatch is directionally asymmetric: hospital and clinic datasets correspond to 2020, whereas pharmacy data reflect the situation in 2025. Support for the reorganization of hospital bed functions has been promoted toward the realization of the regional healthcare vision. In 436 hospitals subject to re‑evaluation, the number of acute care beds is projected to decrease from 40,300 in 2017 to 29,100 in 2025, representing an estimated reduction of approximately 28% [28]. Concurrently, financial pressures have precipitated the closure of smaller public hospitals, particularly in low-density rural areas. The number of hospital beds was 1.246 million in 2018, but is estimated to decrease to 1.218 million by 2025 [29]. Consequently, the actual current hospital coverage rates in these regions may be lower than those estimated in this study.” (page 25 lines 447–456)

New References

28. Ministry of Health, Labour and Welfare. Status of Regional Discussions and Initiatives Related to the Regional Healthcare Vision. 2021. Available from: https://www.mhlw.go.jp/content/10800000/000862586.pdf [cited 2026 Jun 19].

29. Ministry of Health, Labour and Welfare. About the Regional Healthcare Vision. 2020. Available from: https://www.mhlw.go.jp/conten

Attachment Submitted filename: Response to Reviewer 5.docx

# Peer review: Decision Letter 3

**DOI:** 10.1371/journal.pone.0356748.r007

**Authors:** Lingye Yao

6 Aug 2026

Accessibility to medical institutions in Aichi Prefecture: A geographic information systems-based analysis

PONE-D-25-46566R3

Dear Dr. Nakamura,

We’re pleased to inform you that your manuscript has been judged scientifically suitable for publication and will be formally accepted for publication once it meets all outstanding technical requirements.

Within one week, you’ll receive an e-mail detailing the required amendments. When these have been addressed, you’ll receive a formal acceptance letter and your manuscript will be scheduled for publication.

An invoice will be generated when your article is formally accepted. Please note, if your institution has a publishing partnership with PLOS and your article meets the relevant criteria, all or part of your publication costs will be covered. Please make sure your user information is up-to-date by logging into Editorial Manager at Editorial Manager® and clicking the ‘Update My Information' link at the top of the page. For questions related to billing, please contact billing support.

If your institution or institutions have a press office, please notify them about your upcoming paper to help maximize its impact. If they’ll be preparing press materials, please inform our press team as soon as possible -- no later than 48 hours after receiving the formal acceptance. Your manuscript will remain under strict press embargo until 2 pm Eastern Time on the date of publication. For more information, please contact onepress@plos.org.

Kind regards,

Lingye Yao, Ph.D.

Academic Editor

PLOS One

Additional Editor Comments (optional):

Please ensure that the three minor comments from Reviewer #2 are carefully addressed during the proofreading period prior to official publication.

Reviewers' comments:

Reviewer's Responses to Questions

**Comments to the Author**

Reviewer #2: All comments have been addressed

Reviewer #4: All comments have been addressed

**********

2. Is the manuscript technically sound, and do the data support the conclusions?

Reviewer #2: Yes

Reviewer #4: Yes

**********

3. Has the statistical analysis been performed appropriately and rigorously?

Reviewer #2: Yes

Reviewer #4: Yes

**********

4. Have the authors made all data underlying the findings in their manuscript fully available?

The PLOS Data policy

Reviewer #2: (No Response)

Reviewer #4: Yes

**********

5. Is the manuscript presented in an intelligible fashion and written in standard English?

Reviewer #2: Yes

Reviewer #4: Yes

**********

Reviewer #2: The manuscript is now much improved, and several of my previous comments have been addressed. The authors now define accessibility more carefully as proximity-based potential access, include sensitivity analyses using different buffer sizes, acknowledge the limitations of areal weighting, discuss the differences in reference years across datasets, provide a more cautious interpretation of car ownership, and better limit the generalization of the findings. In its current form, I believe the manuscript is suitable for publication.

However, I would like to offer a few minor recommendations for the authors’ consideration.

First, please consider removing the claim that the findings from Aichi Prefecture can be used to infer healthcare access trends in other regions. The manuscript itself acknowledges that external validity or transportability is not guaranteed. Therefore, the statement about “inferring trends” elsewhere is not fully consistent with the limitations section. It would be more appropriate to state that the analytical method may be applied in other regions to examine whether similar patterns are found.

Second, please consider replacing the term “public transport accessibility” with “proximity to transit stops and stations,” “transit-node proximity,” or another more precise term. The analysis only measures whether residents live within 400 m of a bus stop or railway station. As the authors have already acknowledged, residents classified as “covered” may not have a route that actually connects them to a medical institution. Therefore, the term “accessibility” remains too broad for this indicator.

Third, please consider further softening the claim that car ownership compensates for limited healthcare access and, where possible, use more appropriate indicators such as household vehicle ownership rates, driving licence ownership, or modal share. Differences in coverage between buffer sizes only show changes in potential geographic reach under distance assumptions. They do not demonstrate that every resident owns, can drive, or has access to a car. The manuscript itself notes the decline in driving ability among older people and that approximately 25% of older adults do not hold a driving licence.

Apart from these minor points, I believe that all sections of the manuscript are now very well developed.

Reviewer #4: In this revision, the authors have addressed all concerns and the manuscript can be accepted for publication.

**********

what does this mean?). If published, this will include your full peer review and any attached files.

If you choose “no”, your identity will remain anonymous but your review may still be made public.

**Do you want your identity to be public for this peer review?**  For information about this choice, including consent withdrawal, please see our Privacy Policy

Reviewer #2: No

Reviewer #4: No

**********

# Editor report: Acceptance letter

**DOI:** 10.1371/journal.pone.0356748.r008

**Authors:** Lingye Yao

PONE-D-25-46566R3

PLOS One

Dear Dr. Nakamura,

I'm pleased to inform you that your manuscript has been deemed suitable for publication in PLOS One. Congratulations! Your manuscript is now being handed over to our production team.

At this stage, our production department will prepare your paper for publication. This includes ensuring the following:

* All references, tables, and figures are properly cited

* All relevant supporting information is included in the manuscript submission,

* There are no issues that prevent the paper from being properly typeset

You will receive further instructions from the production team, including instructions on how to review your proof when it is ready. Please keep in mind that we are working through a large volume of accepted articles, so please give us a few days to review your paper and let you know the next and final steps.

Lastly, if your institution or institutions have a press office, please let them know about your upcoming paper now to help maximize its impact. If they'll be preparing press materials, please inform our press team within the next 48 hours. Your manuscript will remain under strict press embargo until 2 pm Eastern Time on the date of publication. For more information, please contact onepress@plos.org.

You will receive an invoice from PLOS for your publication fee after your manuscript has reached the completed accept phase. If you receive an email requesting payment before acceptance or for any other service, this may be a phishing scheme. Learn how to identify phishing emails and protect your accounts at https://explore.plos.org/phishing.

If we can help with anything else, please email us at customercare@plos.org.

Thank you for submitting your work to PLOS One and supporting open access.

Kind regards,

PLOS ONE Editorial Office Staff

on behalf of

Dr. Lingye Yao

Academic Editor

PLOS One
